# Integrative analysis of genome-wide association studies identifies novel loci associated with neuropsychiatric disorders

**DOI:** 10.1038/s41398-020-01195-5

**Published:** 2021-01-21

**Authors:** Xueming Yao, Joseph T. Glessner, Junyi Li, Xiaohui Qi, Xiaoyuan Hou, Chonggui Zhu, Xiaoge Li, Michael E. March, Liu Yang, Frank D. Mentch, Heather S. Hain, Xinyi Meng, Qianghua Xia, Hakon Hakonarson, Jin Li

**Affiliations:** 1 Department of Cell Biology, the Province and Ministry Co-sponsored Collaborative Innovation Center for Medical Epigenetics, School of Basic Medical Sciences, Tianjin Medical University, Tianjin, China; 2 Center for Applied Genomics, the Children’s Hospital of Philadelphia, Philadelphia, PA USA; 3 Department of Endocrinology and Metabolism, Tianjin Medical University General Hospital, Tianjin, China; 4 Department of Pediatrics, Jinnan Hospital, Tianjin, China; 5 Center for International Collaborative Research on Environment, Nutrition and Public Health, Tianjin Key Laboratory of Environment, Nutrition and Public Health, School of Public Health, Tianjin Medical University, Tianjin, China; 6 Department of Nutrition and Food Science, School of Public Health, Tianjin Medical University, Tianjin, China; 7 Division of Human Genetics, The Children’s Hospital of Philadelphia, Philadelphia, PA USA; 8 Department of Pediatrics, The Perelman School of Medicine, University of Pennsylvania, Philadelphia, PA USA; 9 Tianjin Eye Hospital, Tianjin, China; 10 Tianjin Key Laboratory of Ophthalmology and Visual Science, Tianjin Eye Institute, Tianjin, China

**Keywords:** ADHD, Personalized medicine

## Abstract

Neuropsychiatric disorders, such as autism spectrum disorder (ASD), attention deficit hyperactivity disorder (ADHD), schizophrenia (SCZ), bipolar disorder (BIP), and major depressive disorder (MDD) share common clinical presentations, suggesting etiologic overlap. A substantial proportion of SNP-based heritability for neuropsychiatric disorders is attributable to genetic components, and genome-wide association studies (GWASs) focusing on individual diseases have identified multiple genetic loci shared between these diseases. Here, we aimed at identifying novel genetic loci associated with individual neuropsychiatric diseases and genetic loci shared by neuropsychiatric diseases. We performed multi-trait joint analyses and meta-analysis across five neuropsychiatric disorders based on their summary statistics from the Psychiatric Genomics Consortium (PGC), and further carried out a replication study of ADHD among 2726 cases and 16299 controls in an independent pediatric cohort. In the multi-trait joint analyses, we found five novel genome-wide significant loci for ADHD, one novel locus for BIP, and ten novel loci for MDD. We further achieved modest replication in our independent pediatric dataset. We conducted fine-mapping and functional annotation through an integrative multi-omics approach and identified causal variants and potential target genes at each novel locus. Gene expression profile and gene-set enrichment analysis further suggested early developmental stage expression pattern and postsynaptic membrane compartment enrichment of candidate genes at the genome-wide significant loci of these neuropsychiatric disorders. Therefore, through a multi-omics approach, we identified novel genetic loci associated with the five neuropsychiatric disorders which may help to better understand the underlying molecular mechanism of neuropsychiatric diseases.

## Introduction

Neuropsychiatric disorders, such as autism spectrum disorder (ASD), attention deficit hyperactivity disorder (ADHD), schizophrenia (SCZ), bipolar disorder (BIP), and major depressive disorder (MDD) are a spectrum of common, complex, polygenetic diseases^1–4^. Neuropsychiatric disorders have become a major public health concern. It is estimated that every year 38.2% of the European population (164.8 million persons) are suffering from a neuropsychiatric disorder^5^. Some neuropsychiatric disorders, such as the common childhood behavioral disorders ASD and ADHD, are characterized by the early age of onset^6,7^. For example, one in 69 children aged 8 suffer from ASD^6^. The average age at diagnosis for ADHD is 7 years old and symptoms can appear as early as 3 years old^8^.

It is often hard to define clear clinical boundaries between these diseases. MDD, SCZ, and BIP share frequent clinical symptoms, such as anxiety, psychosis, mood swings, and depression^9^. Accumulating evidence suggests a common genetic overlap between neuropsychiatric disorders. Familial co-segregation of SCZ and BIP was observed in a Swedish population study^10^, and the increased cross-disorder risk was found to be associated with a family history of SCZ, mood disorders, ASD, and ADHD^11^. These family based studies demonstrated the co-inheritance of neuropsychiatric disorders. Early onset MDD was found to have genetic overlap with SCZ and BIP based on polygenic risk score analysis^12^. Thus, it is important to understand the common and unique genetic and pathogenic features of these diseases, which may be of help to design efficient and effective treatments.

Each of these five neuropsychiatric disorders is highly heritable, with heritability estimates in twin studies ranging from 40 to 88%^13–20^, and a number of genome-wide significant (GWS) loci have been identified from large scale genome-wide association studies (GWASs), especially through the collective effort of the Psychiatric Genomics Consortium (PGC). Among the five neuropsychiatric disorders, SCZ has been best studied with ~200 GWS loci identified as summarized in the GWAS catalog^21,22^, whereas the number of GWS loci for MDD and BIP are somewhat less with 44 and 30 discovered loci, respectively, based on recent studies^23,24^. ADHD and ASD are disorders with the least number of GWS loci reported, though they are of similar prevalence and heritability as SCZ. GWS loci have been discovered in recent studies for ADHD and ASD by PGC^25,26^ with increased case numbers of over 15,000 for each disease. As common variants usually only confer a small effect on the disease risk, a large sample size is necessary for GWS locus identification. All the largest GWAS of each of these neuropsychiatric disorders to date were carried out by PGC ranging from 18,381 cases and 27,969 controls for ASD to 135,458 cases and 344,901 controls for MDD (including iPSYCH samples in the MDD cohort)^21,23–26^. Additional loci were found when trans-ancestry GWAS was conducted. A meta-analysis of SCZ in the largest combined cohort of 56,418 cases and 78,818 controls including both European ancestry and East Asian subjects generated 53 novel loci^21^. The single-nucleotide polymorphism (SNP)-based heritability (*h*_G_^2^) was estimated to be within the range of 8.7% (MDD) to 24% (SCZ) in these large-scale GWASs, still much lower than the heritability estimated from twin studies. Many genetic loci have been shown to be pleiotropic, associated with multiple related traits or disorders. For example, the well-known locus of gene *GRIN2A* was associated with SCZ^21^ and BIP^23^; the newly identified locus of *SEMA6D* was associated with both ADHD^25^ and MDD^27^. To identify additional loci associated with neuropsychiatry disorders with increased study power and to systematically examine pleiotropy of loci associated with neuropsychiatric disorders, cross-cohort meta-analyses have been conducted for the PGC cohorts. An early PGC study that meta-analyzed a cohort of 33,332 cases with the diagnosis of ADHD, ASD, BIP, MDD, or SCZ and 27,888 controls yielded the identification of 4 GWS loci with pleiotropic effects^28^. The recent meta-analysis of 8 neuropsychiatric disorders by PGC further reported 109 pleiotropic loci, including 23 associated with four or more disorders and 11 showing differential directions of effects between neuropsychiatric disorders with an increased sample size of 232,964 cases and 494,162 controls^29^. These studies have made significant advances in our understanding of the genetic architecture and molecular mechanism of neuropsychiatric disorders. To take advantage of recent improvements in technical and analytical methods, we conducted a meta-analysis of five correlated neuropsychiatric disorders of ADHD, ASD, BIP, MDD, or SCZ and a joint-analysis using the multi-trait analysis of GWAS (MTAG) method which has been shown to increase statistical power to identify genetic loci associated with each individual disease by incorporating information from other correlated traits. We further performed a replication study in an independent pediatric cohort for the novel loci of ADHD which usually presents as a childhood-onset neurodevelopmental disorder. Our study replicated multiple of the discoveries reported in the recent cross-cohort meta-analysis, using a complementary approach and further identified novel loci.

## Methods

### GWAS data of neuropsychiatric disorders

GWAS summary statistics were downloaded from the website of the PGC (https://www.med.unc.edu/pgc). All five largest cohorts of European ancestry for the five neuropsychiatric disorders of ADHD^25^, ASD^26^, BIP^23^, MDD^30^, and SCZ^21^ were selected, which are listed in Supplementary Table 1 and described in detail below.

#### ADHD|PMID: 30478444

This is a meta-analysis including 12 cohorts of European, North American, and Chinese ancestry collected by the Lundbeck Foundation Initiative for Integrative Psychiatric Research (iPSYCH) and PGC, which contained 20,183 ADHD cases and 35,191 controls. The cases of iPSYCH were diagnosed by psychiatrists in accordance with ICD10 (F90.0) and genotyped using Illumina PsychChip while the cases of the remaining cohorts were diagnosed in hospitals or clinics by professional clinicians. Here we used the summary statistics of a meta-analysis of the European ancestry subjects composed of 19,099 cases and 34,194 controls.

#### ASD|PMID: 30804558

Data from a Danish population-based cohort and five family based cohorts of trio samples of European ancestry were utilized in the meta-analysis of this publication. The cases with ASD were diagnosed according to the expert clinical consensus or ICD-10. Summary statistics from meta-analysis of all the samples, including 18,381 cases and 27,969 controls, were included in our analysis.

#### BIP|PMID: 31043756

The meta-analysis is composed of 32 cohorts of European descent, including 20,352 cases and 31,358 controls. Diagnoses were confirmed with the international consensus criteria of DSM-IV, ICD-9, or ICD-10. The meta-analysis summary statistics from all subjects were utilized in our study.

#### MDD|PMID: 30718901

This meta-analysis combined three previous studies of depression from UK Biobank, 23andme, and PGC, including a total of 246,363 cases and 561,190 controls. As reported in the original meta-analysis, the determination of case or control status of the subjects in the UK Biobank dataset was mostly self-reported, and the case status of samples in the PGC dataset was determined by structured clinical interviews or medical records. Due to the unavailability of full summary statistics of 23andme data, we only used the summary level data from PGC and UK Biobank with 170,756 cases and 329,443 controls.

#### SCZ|PMID: 31740837

The study is a meta-analysis of East Asian subjects (22,778 cases and 35,362 controls) and European descent subjects (33,640 cases and 43,456 controls). The summary statistics of European descent subjects were derived from Ripke et al.’s work^31^ and updated to 1000 genomes phase 3. We only used the summary statistics of European descent subjects in our study.

### Multi-trait joint analysis

Joint analysis of the GWAS summary statistics was carried out using software MTAG^32^, which takes disease heterogeneity into account. The alternative allele frequency information of each SNP was taken from 1000 G data of European ancestry^33^.

### Meta-analysis

A meta-analysis based on the summary statistics of the above five neuropsychiatric diseases was conducted with the *P* value-based method implemented in the software METAL with sample overlap correction^34^.

### Defining independent genomic loci

PLINK was used to clump the GWS SNPs into linkage disequilibrium (LD)-independent genomic regions (–clump-r2 = 0.4, –clump-kb = 500, –clump-p1 = 5e-08, –clump-p2 = 5e-02)^35^. LD information from the 1000 G data of European ancestry was applied in clumping. Genomic loci with the distance between index SNPs less than 500 kb were merged. The MHC region (chr6:25–35 MB) was regarded as one locus owing to the extensive LD.

### Determination of novel loci

The novel loci were determined in the following four steps by excluding known loci. (a) The results obtained for each neuropsychiatric disorder through MTAG analysis were clumped with the reported associations of each corresponding disease in GWAS Catalog^36^ using PLINK, and the results from the METAL analysis were clumped with the reported associations of all five diseases in GWAS Catalog. The parameters and LD information used here were the same as those used in the step of defining independent genomic loci. All GWS SNPs located in the same clump with the significant SNPs reported in the GWAS catalog were considered as known SNPs, and the loci containing these SNPs were considered as known loci. (b) All the significant loci reported in the original GWAS article of each neuropsychiatric disorder and the recent meta-analysis of eight neuropsychiatric disorders by PGC were considered as known loci. (c) GWS SNPs from our analyses located within a 1 MB range of a reported significant locus from the above two resources were considered to be within known loci. (d) If a locus containing GWS SNPs in our analyses was merged by stepwise conditional analysis with a known locus using GCTA-COJO^37^, the locus was regarded as known. The GWS loci left after the above exclusion steps were defined as novel loci.

### Fine mapping of causal variants

A stepwise conditional analysis was further performed using software package GCTA-COJO to identify the independent associations at each locus, based on LD information from the 1000 G EUR reference panel. Fine mapping of causal variants at each novel locus was conducted using the software FINEMAP^38^ with the setting of five as the maximum number of allowed causal SNPs. The required input of LD file was also generated from PLINK with a 1000 G EUR reference panel. For each novel locus, SNPs in a 95% credible set with posterior probabilities of 1, in LD with the index SNP (*r*^2^ > 0.6) and with MTAG association *P* value < 1 × 10^−4^ were selected as the potential causal variants.

### Estimation of SNP-based heritability and genetic correlation

The SNP-based heritability on the liability scale and genetic correlation of these neuropsychiatric disorders were estimated using the software LD score (LDSC)^39^ based on the MTAG analysis results. Precomputed LDSC from 1000 G European ancestry samples were used in both analyses with population prevalence for each trait being consistent with that reported in the original publication of each cohort listed in Supplementary Table 1. Besides, partitioning of the SNP-based heritability by functional category using the baseline model was conducted for 53 functional overlapping annotations^40^ with the MTAG summary statistics. The baseline model LDSC, regression weights, and allele frequencies computed from 1000 G phrase 3 data were downloaded from the website http://data.broadinstitute.org/alkesgroup/LDSCORE/.

### Functional annotation

Functional annotation of MTAG results was conducted through web portal FUMA^41^. The mapping of SNPs to corresponding candidate genes took into account the distance between SNPs and genes, expression quantitative trait loci (eQTLs), and the long-range chromatin interactions (Hi-C) between SNPs and genes. The eQTL data sets used in our analysis were PsychENCODE^42^, BRAINEAC^43^, and GTEx v8^44^ Brain; and Hi-C data specifically from brain tissues and cell types were used in our analysis, including data from PsychENCODE, adult and fetal brain, as well as neural progenitor cell^42,45^.

### Histone modification site analysis

Histone modification analysis was conducted via web portal Haploreg and the overlap between histone modification regions and potential causal variants was evaluated based on epigenome data in the brain from epigenomics databases of ROADMAP project^46,47^.

### Gene set enrichment and gene expression profile analyses

The gene set enrichment analysis and gene expression profile analysis among all the significant loci in MTAG results for each disease were performed via MAGMA^48^ implemented in FUMA. The resources of gene sets were from predefined MSigDB^49^ Gene Ontology gene sets with 5497 curated gene sets and 9986 GO terms. The significant enrichment of gene sets was based on hypergeometric tests and the significance threshold was set as *P* value < 0.05 after multiple-testing adjustment. The gene expression profile analysis was also carried out through FUMA with the Brainspan dataset which contains reads per kilobase of transcript per million mapped reads (RPKM) values of 21,001 genes for brain samples of 29 different ages and 19,601 genes for 11 general developmental stages^50^.

## GWAS replication study

### Study subjects

The replication study was conducted at the Center for Applied Genomics (CAG), the Children’s Hospital of Philadelphia (CHOP). The ICD codes used to retrieve the ADHD cases are listed in Supplementary Table 2. For controls, we included subjects without ADHD diagnosis or any other relevant neuropsychiatric conditions. The age range for subjects in our study is from 3 to 21 years old with an average age of 6.

### Ethics statement

The study was approved by the Institutional Review Board at CHOP and all participants provided written informed consent. The replication cohort is independent of the PGC cohort.

### Genotyping

Genomic DNA of the samples in the replication study was extracted from whole blood or saliva samples following the phenol–chloroform protocol and genotyped on Illumina HumanHap550-V1/V3, HumanHap610-Quad, or Infinium Global Screening Array-24 (GSA) DNA Bead Chips at CAG.

### Quality control (QC) filtering

QC steps were performed with PLINK (software release v1.9)^35^. Sample QC includes the following criteria. Samples with call rate <98%, or with ambiguous sex detected by PLINK as well as heterozygosity outliers were excluded. Duplicated and cryptically related samples were identified as PI_HAT ≥ 0.1875 in PLINK identity-by-state analysis, and one from each pair was excluded. SNP based QC was conducted according to the following measurements. SNPs with genotype rate <95%, minor allele frequency <0.01, or Hardy–Weinberg equilibrium *P* values < 10^−5^ were removed from further analysis.

### Principal component analysis (PCA)

PCA was conducted with PLINK and samples that were not of European ancestry were excluded based on PCA plots. Afterward, PCA was performed again to derive the principal components which were used as covariates to control for population stratification.

A total of 5194 ADHD cases and 33,127 controls were genotyped, among which, 2726 ADHD cases and 16,299 healthy controls of European ancestry, with 495,535 variants shared on the V1/V3Quad arrays and 497203 variants on the GSA arrays passing QC.

### Genome-wide imputation

For all samples genotyped on different platforms, unphased GWAS genotype data were converted to VCF files and uploaded to Michigan Imputation Server (https://imputationserver.sph.umich.edu/) for phasing using Eagle2^51^ and imputation using Minimac3^52^ with the Haplotype Reference Consortium reference panel. Afterward, variants with imputation score *R*^2^ < 0.4 were filtered out. Further, after SNP QC described above, 7,617,389 variants shared across genotyping platforms were included in the association testing.

### Association analysis

After imputation and post-imputation quality control filtering, logistic regression was conducted to evaluate the association between SNP genotype and neuropsychiatric disease status, including sex and the first ten principal components as covariates.

### Sign test

Summary statistics of overlapping SNPs in both the ADHD MTAG analysis and the pediatric replication cohort were extracted. PLINK clumping and GCTA-COJO step-wise conditional analyses were similarly performed to identify independent associations as described above, with the European ancestry population of 1000 Genomes as reference. The sign test was performed to compare the direction of effect at each GWS association between the ADHD MTAG analysis results and our pediatric replication cohort. For all the GWS associations (*P* value < 5 × 10^−8^) in the ADHD MTAG analysis, the proportion of concordant direction of effect (*π*) in the replication cohort was tested against a null hypothesis of *π* = 0.50 based on the one-sample test of the proportion with Yates’ continuity correction.

### Test for replication of effect size

Adjustment for winner’s curse was first performed on the ADHD MTAG results via the method of FDR Inverse Quantile Transformation (FIQT)^53^. Then for all the GWS associations in the ADHD MTAG analysis, the effect size in the replication cohort was regressed on the adjusted effect size from the ADHD MTAG analysis. The slope of the regression was tested against the null hypothesis of the expected slope being zero or one respectively.

### Test for genetic correlation of the replication cohort

The genetic correlation between the ADHD MTAG analysis and our replication cohort was similarly computed using LDSC regression as described above for the genetic correlation analyses between neuropsychiatric disorders based on genome-wide data.

## Results

### Joint analysis of GWAS summary statistics

To identify novel genetic factors associated with neuropsychiatric diseases, we conducted a multi-trait joint analysis based on summary statistics of the five neuropsychiatric GWASs of ADHD, ASD, BIP, MDD, and SCZ (Supplementary Table 1), taking phenotype correlation into account. The standardized genomic inflation factor for MTAG analysis of each neuropsychiatric disease was close to one, suggesting no evidence of inflation due to confounding (Supplementary Fig. 1). A number of loci reached the genome-wide significance (GWS) threshold for each disease, including 20 for ADHD, 9 for ASD, 54 for BIP, 74 for MDD, and 90 for SCZ, respectively (Supplementary Table 3). We identified 16 novel GWS loci, including 5 loci for ADHD, 1 for BIP, and 10 for MDD, which have not previously been reported to be associated with the corresponding neuropsychiatric disorder at the GWS level (Table 1, Supplementary Fig. 2, Supplementary Table 4). We also found SNPs at other loci in each of the MTAG results overlapped with the GWS loci reported in the recent meta-analysis of eight neuropsychiatric disorders^29^ (9 for ADHD, 7 for ASD, 49 for BIP, 25 for MDD, and 77 for SCZ) (Supplementary Table 5). While this reduced the total number of novel GWS loci found in our MTAG analyses, it demonstrated the reliability of our analyses, being consistent and complementary to the meta-analysis with the approach of association analysis based on SubSETs (ASSET). All of the novel loci identified showed evidence of association (*P* < 0.05) with two or more neuropsychiatric disorders and the direction of effects were consistent across the diseases (Supplementary Fig. 3, Supplementary Table 4). Of note, eleven of the 16 novel GWS loci exhibited consistent genome-wide marginal significance (*P* < 5 × 10^−5^) between ADHD and MDD. The locus at 18q12.3 reached GWS for the first time for both ADHD and MDD. Such concordance between ADHD and MDD is in line with recent evidence showing a strong genetic correlation between ADHD and MDD^25^, as well as the high level of comorbidity between ADHD and MDD. We noticed that genetic variants, including SNPs and CNVs, at several of the novel loci have been found to be associated with other neuropsychiatric or neurodegeneration diseases, including variants in gene *IP6K1* which are associated with general cognitive ability^54^, educational attainment^55^, and intelligence^56^; variants at the *TRAF3* locus which are associated with brain imaging in SCZ at genome-wide marginal significance level^57^; and mutations in *SPTBN2* that cause spinocerebellar ataxia with neurodegenerative feature^58^.

**Table 1:** Novel genome-wide significant loci from multi-trait joint-analyses of neuropsychiatric disorders.

Disease	Locus	Chr	POS(hg19)	Lead SNP	Cytoband	Gene	A1	A2	Freq	Beta	SE	*P*
ADHD	1	3	49809841	rs9870755	3p21.31	IP6K1	T	C	0.16	0.090	0.016	6.14E−09
	2	5	92995013	rs71639293	5q15	FAM172A	A	G	0.81	0.090	0.016	2.53E−08
	3	11	49356806	rs10839264	11p11.12	FOLH1	T	C	0.08	0.118	0.021	2.27E−08
	4	18	39300226	rs8083506	18q12.3	PIK3C3,KC6	T	C	0.21	−0.089	0.015	6.19E−09
	5	20	44730245	rs6032660	20q13.12	NCOA5	A	G	0.74	−0.083	0.014	2.77E−09
BIP	6	1	95578207	rs12563424	1p21.3	TMEM56	T	C	0.64	−0.064	0.011	1.29E−08
MDD	7	1	191027948	rs10920885	1q31.2	RP11-309H21.3, LOC440704	T	G	0.81	−0.032	0.005	2.26E−09
	8	3	29756378	rs9834021	3p24.1	RBMS3	T	C	0.49	−0.023	0.004	3.18E−08
	9	7	114059156	rs2894699	7q31.1	AC073626.2, FOXP2	T	C	0.43	−0.025	0.004	4.15E−09
	10	11	28642653	rs4636654	11p14.1	RP11-960D24.1, METTL15	A	G	0.40	−0.027	0.004	2.83E−10
	11	11	66456387	rs2276138	11q13.2	SPTBN2	T	C	0.48	−0.023	0.004	2.18E−08
	12	13	97208898	rs1414622	13q32.1	HS6ST3	T	C	0.92	0.043	0.008	3.03E−08
	13	14	103301072	rs7146581	14q32.32	TRAF3	T	C	0.22	0.027	0.005	2.3E−08
	14	15	66529936	rs4776768	15q22.31	MEGF11	T	C	0.28	−0.025	0.005	3.71E−08
	15	18	39296488	rs8099746	18q12.3	PIK3C3,KC6	T	C	0.80	0.030	0.005	1.27E−08
	16	22	46457723	rs62228096	22q13.31	hsa-mir-4763, LOC150381	A	G	0.35	−0.026	0.004	4.84E−09

*Disease* the name of the neuropsychiatric disorders, *ADHD* attention deficit hyperactivity disorder, *BIP* bipolar disorder, *MDD* major depressive disorder, *CHR* chromosome, *POS* the position of each SNP on the human genome build hg19, *SNP* the most significantly associated SNP at each locus, *Gene* closest gene, *A1* the effect allele, *A2* the non-effect allele, *Beta* the association coefficient, *Freq* the frequency of the effect allele, *SE* the standard error of the effect size estimate; *P P* value in MTAG analysis.

### A meta-analysis of GWAS summary statistics of five neuropsychiatric disorders

**Fig. 1:**
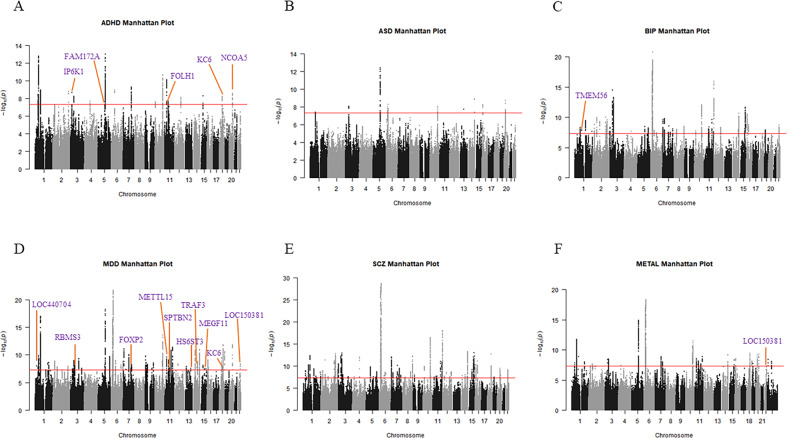
Manhattan plots showing the association statistics for multi-trait joint analysis and meta-analysis of five neuropsychiatric diseases. Manhattan plots of the susceptibility loci of five neuropsychiatric diseases: ADHD (**A**), ASD (**B**), BIP (**C**), MDD (**D**), SCZ (**E**), METAL (**F**). SNP locations are plotted on the *x*-axis according to their chromosomal position. The *y*-axis shows the −log_10_ of *P* values per SNP derived from the meta-analysis. The horizontal red line represents the genome-wide significance threshold of *P* value < 5 × 10^−8^. The purple text shows the closest gene to the most prominent SNPs at each novel locus.

We further conducted a meta-analysis based on PGC GWAS summary statistics of the five neuropsychiatric disorders using the software METAL, which yielded 3095 GWS SNPs (*P* value < 5 × 10^−8^) located at 43 loci, including one novel locus. This novel locus on chr22 has also reached GWS in the MTAG analysis of MDD (Table 2, Fig. 1, Supplementary Figure 4, Supplementary Table 6).

**Table 2:** Novel locus from a meta-analysis of five neuropsychiatric disorders.

Chr	POS	SNP	A1	A2	META_P	Direction	Gene	Disease	Beta	SE	P
22	46457723	rs62228096	A	G	4.48E-08	–––––	hsa-mir-4763, LOC150381	ADHD	−0.022	0.014	3.42E−04
								ASD	−0.019	0.014	2.47E−03
								BIP	−0.005	0.014	4.22E−01
								MDD	−0.023	0.005	6.38E−07
								SCZ	−0.018	0.012	2.82E−04

*Chr* chromosome, *POS* the position of each SNP on the human genome build hg19, *SNP* the most significantly associated SNP at each locus, *A1* the effect allele, *A2* the reference allele, *META_P* *P* value in the meta-analysis, *Direction* summary of effect direction for each study, with one “+” or “−” per the study, and “+” indicates A1 allele confers risk effect while “−” indicates A1 allele confers protective effect, *Gene* closest gene; *disease* the name of five neuropsychiatric disorders, *ADHD* attention deficit hyperactivity disorder, *ASD* autism spectrum disorder, *BIP* bipolar disorder, *MDD* major depression disorder, *SCZ* schizophrenia, *Beta* the association coefficient in the original summary statistics of each disease-specific cohort, *SE* the standard error of the effect size estimate in original summary statistics of each disease-specific cohort, *P P* value in the original summary statistics of each disease-specific cohort.

### Fine mapping and biological annotation of causal variants at novel GWS loci

To understand how the novel loci of ADHD, BIP, and MDD contribute to the development of neuropsychiatric disorders, we conducted fine-mapping to find the likely causal variants at each locus. We found multiple potential causal variants at each locus with the marginal posterior inclusion probabilities of one and the association *P* value in meta-analysis less than 5 × 10^−5^. The results showed that the index SNP at each locus serves as one of the potential causal variants. The detailed information for each locus is shown in Supplementary Table 7.

We further performed functional annotation on the likely causal variants at each novel locus identified, examining the involvement of these SNPs in chromatin interactions, histone modification, and eQTL effects in brain tissues. Among the likely causal variants, 20 overlap with histone marks at gene enhancer or promoter regions in brain tissues (Supplementary Table 8). A total of 22 causal SNPs are brain eQTL SNPs in different brain regions (Supplementary Table 9). In addition, 12 loci showed chromatin interactions with distant genes (Supplementary Fig. 5, Supplementary Table 10). Therefore, multiple lines of evidence support the role of the potential causal SNPs at these novel loci in regulating gene expression.

### Gene expression profile and gene set enrichment analysis

Gene expression profile analysis of genes at all the novel loci demonstrated the enrichment of differentially expressed gene sets at early developmental stages (Supplementary Fig. 6).

We also conducted a gene-set analysis to gain insights into the pathways involving genes at GWS loci of neuropsychiatric disorders. We found that the GWS loci of each disorder were notably enriched in genes in GO cellular component of a postsynaptic membrane or postsynaptic density membrane, which was consistent with other reports on the involvement of postsynaptic components in the pathology of neuropsychiatric disorders^59^. In addition, GWS loci were also enriched in GO biological functions of synaptic signaling, membrane depolarization, neuron differentiation for particular neuropsychiatric disorders (Supplementary Table 11). Our results indicated the highly significant enrichment of GWS loci of neuropsychiatric disorders in various aspects of neuronal activity.

### The SNP-based heritability and genetic correlation of neuropsychiatric disorders

To evaluate the contribution of common variants to the phenotypic variance of each neuropsychiatric disorder, we conducted a SNP-based heritability estimation based on MTAG results. The SNP-based heritability on the liability scale to each disease ranged from 0.109 to 0.382 (ADHD *h*^2^_SNP_ = 0.339, ASD *h*^2^_SNP_ = 0.217, BIP *h*^2^_SNP_ = 0.382, MDD *h*^2^_SNP_ = 0.109, SCZ *h*^2^_SNP_ = 0.241), slightly increased when compared to what was reported in the cohort’s original publication, with consistent population prevalence (Supplementary Fig. 7); however, SNP-based heritability was still much less than that estimated from family based or population-based samples (Supplementary Table 12). Further analyses partitioning SNP-based heritability showed significant enrichment in the *h*^2^_SNP_ from SNPs at the functional category of Conserved LindbladToh in all the five neuropsychiatric disorders, which implicated the eminent contribution of conserved regions to the SNP-based heritability of neuropsychiatric diseases. We also observed significant *h*^2^_SNP_ partitioning to the categories of H3K9ac_peaks and H3K4me1 across five disorders, suggesting that these neuropsychiatric GWS loci may be involved in disease pathogenesis through transcriptional regulation (Supplementary Table 13).

We also assessed the relationship between these neuropsychiatric disorders by LDSC regression analyses and observed a highly significant genetic correlation between them (Supplementary Fig. 8), which is consistent with the reported estimates in previous studies by PGC^29^. Among them, SCZ and BIP showed the highest genetic correlation, followed by that between MDD and ADHD which was also reflected by the shared novel loci between them (Table 1, Supplementary Table 4).

### The replication study

Among the neuropsychiatric disorders which yielded GWS signals in our MTAG analyses, ADHD is less studied than BIP and MDD. ADHD is an early onset childhood disease; it has been reported that almost all patients with clinical features of ADHD retrospectively recalled an onset before age 16. We are, therefore, particularly interested in understanding the genetic mechanism behind ADHD. We conducted a replication study on 2726 ADHD cases and 16,299 healthy controls of European ancestry in a pediatric cohort at CAG, CHOP, which was genotyped on three GWAS chip types. We conducted genome-wide imputation and association testing with no evidence of population stratification (*λ* = 1.013, Supplementary Fig. 9). Due to the limited number of ADHD cases in the replication cohort, we only observed modest associations. Among the five novel loci for ADHD in the MTAG analysis, the lead SNPs at two loci were of nominal association *P* value < 0.05 in the replication study, including rs10839264 at 11p11.12 (*P* value = 0.0487), rs8083506 at18q12.3 (*P* value = 0.0253); and rs6032660 at 20q13.12 is of marginal significance with *P* value = 0.0528 (Supplementary Table 14).

We further conducted a sign test on all the ADHD GWS associations and observed sign concordance significantly higher than expected by chance (*π* = 75% concordant; *P* = 6.27 × 10^−3^) between the pediatric replication cohort and the ADHD MTAG analysis results. We further tested the replication of the effect sizes of the ADHD GWS associations from MTAG analysis in the replication cohort by regressing the effect size in the replication cohort on the estimated effect size from the ADHD MTAG analysis adjusted for winner’s curse (Supplementary Fig. 10). The result demonstrated a significantly positive slope (slope = 0.645, *P* = 2.28 × 10^−3^) and it did not show a significant difference from 1 (*P* = 0.0735), which suggested that the effect size estimates in the pediatric replication cohort were significantly correlated with the adjusted effect size in the ADHD MTAG analysis. Therefore, though the index SNPs at the novel GWS associations from the ADHD MTAG analysis showed only modest association in the replication cohort due to the sample size limitation, the direction and magnitude of the effect size of all the primary ADHD GWS loci were significantly correlated between the two cohorts. We then examined the genetic correlation of genome-wide data between the ADHD MTAG analysis and the replication cohort and found a strong positive correlation (*r*_g_ = 0.663, SE = 0.183, *P* = 3 × 10^−4^). Considering the average age-of-onset in adulthood for BIP and MDD, the effect size of the GWS loci, and the limited sample size of cases with either of these two disorders in our pediatric cohorts, they are not suitable for replication study of MTAG analysis results for BIP and MDD.

## Discussion

In our study, we conducted an integrative analysis of five common and comorbid neuropsychiatric disorders. Through a joint analysis based on correlated traits and fixed-effect meta-analysis, we identified 16 novel GWS loci associated with one or more diseases. As the index SNPs may just be the tag SNPs for the causal variants, we performed fine-mapping to identify potential causal variants at each locus. Furthermore, the index SNPs and the causal variants are located in the non-coding region of the human genome, likely functioning by regulating target gene expression, therefore we identified potential target genes by positional mapping, regulatory and functional mapping. In cells, the protein products encoded by these target genes do not function alone, instead, they cooperate through protein-protein interaction, and by signaling transduction, so we further performed pathway enrichment and PPI network analysis and found the enrichment of synaptic signaling, membrane depolarization, neuron differentiation among the target genes. We then conducted a replication study with an independent pediatric cohort. The identification of pleiotropic GWS loci between neuropsychiatric disorders may help to understand the shared underlying molecular mechanisms for the design of a common therapeutic strategy and drug repositioning for neuropsychiatric disorders.

In comparison to the recently published cross-disorder meta-analysis of eight neuropsychiatric diseases, we found that many GWS loci obtained from the MTAG analysis overlapped with those reported in the recent meta-analysis^29^. While this reduced the total number of novel GWS loci found in our MTAG analyses, it demonstrated the reliability of our analyses, being consistent and complementary to the meta-analysis with the approach of association analysis based on SubSETs (ASSET). Similarly, the MTAG analysis on intelligence and education by Hill et al.^56^ and the ASSET analysis on cognitive ability, education, and SCZ by Lam et al.^60^ yielded consistent and complementary results, as compared and discussed in the latter study. In addition, our MTAG analysis results explicitly demonstrated the direction and strength of disease-specific association in the analyses with boosted power, compared to the ASSET analysis.

All the novel loci identified in our study are compelling, among which a few loci from ADHD MTAG analysis achieved weak replication as results from an independent pediatric cohort showed moderate sign concordance and correlation of effect size for the GWS loci although none of the P-value surpassed the multiple-testing threshold. The index SNP and the causal variants at locus 11p11.12 are eQTL SNPs for genes *GRM5* and *FOLH1*. *GRM5* encodes metabotropic glutamate receptor 5 belonging to the G protein-coupled receptor family, which adopts a conformation change upon ligand binding, inducing downstream signaling transduction through guanine nucleotide-binding proteins and leading to the activation of down-stream effectors, such as phospholipase C. Accumulating evidence suggests an important function of *GRM5* in regulating synaptic plasticity and neural network activity. The expression of *GRM5* is highly enriched in different brain regions, especially the cerebral cortex. A deletion within *GRM5* has been reported to be found in parents and their children with ADHD^61^ and variants in the *GRM5* gene have been linked to other neuropsychiatric disorders, like SCZ^62,63^. Grm5 knockout mice displayed consistent defects in nerve system and abnormal neurological and behavior phenotypes at different genetic background^64,65^, and similar behavioral deficits have been observed in rats treated with specific negative allosteric modulators of mGluR5^66,67^. GRM5 has been regarded as a promising drug target for neuropsychiatric and neurodegenerative disorders^68,69^. Another eQTL gene for SNPs at ADHD locus 11p11.12 is *FOLH1*, the protein product of which is a type II transmembrane glycoprotein with glutamate carboxypeptidase activity. Its substrates include the nutrient folate and the neuropeptide N-acetyl-l-aspartyl-l-glutamate (NAAG). Both immunohistochemistry and RNAseq data demonstrated an enhanced expression of FOLH1 in intestine and brain tissues, in both the central and peripheral nervous system. The role of *FOLH1* in the brain involves hydrolysis of neuropeptide NAAG to generate glutamate and thus to regulate excitatory neurotransmission^70^. Coding variant H475Y in this gene results in reduced absorption of folates and consequently hyperhomocysteinemia which confers an increased risk for defects in the central nervous system and cognitive function^71^. The genetic variants of *FOLH1* and other genes in the folate metabolism pathway have also been associated with the symptoms of SCZ^72^, in concordance with the epidemiological studies that inadequate maternal folate intake during pregnancy is a risk factor for SCZ^73^.

Another locus that reached GWS for ADHD and was replicated at nominal significance in our replication cohort is 18q12.3, of which the candidate gene is *PIK3C3* (phosphatidylinositol 3-kinase catalytic subunit type 3), regulating degradative endocytic trafficking. Several studies have reported that rare CNVs overlapping with this gene are associated with SCZ^74^ and a variant at the promoter of the *PIK3C3* gene is also associated with increased risk for SCZ and BIP among both European and African American ancestries^75,76^, as well as aberrant DNA methylation level in patients with ASD^77^. In a mouse model, knockout of *Pik3c3* led to abnormal sensory neuron morphology, increased neuron apoptosis and axon degeneration, as well as some dysregulation of the immune system like increased T cell apoptosis, etc^78^.

Several SNPs in high LD with the index SNP rs6032660 at locus 20q13.12 are close to gene *NCOA5* and overlap with the promoter or enhancer histone marks H3K4me1, H3K4me3, H3K27ac, and H3K9ac at various brain regions. Furthermore, they are eQTL SNPs for genes *CD40* and *SLC12A5*. Both *NCOA5*^27,79^ and *SLC12A5*^30,80^ have been reported to be associated with depression. SNPs at the locus of *CD40* are also associated with educational attainment at the GWS level^81^. The protein product of *CD40* is a receptor on antigen-presenting cells, playing critical roles in immune and inflammatory responses. Concordantly, this locus is associated with diverse autoimmune diseases, such as rheumatoid arthritis^82^, multiple sclerosis^83^, and systemic lupus erythematosus (SLE)^84^. *CD40* is therefore a pleiotropic locus for both psychiatric disorders and immune diseases, which supports the notion of a mechanistic link between the immune system and the neural system. In addition, in the early stage of Alzheimer’s disease, CD40 has been reported to play a unique role in the amyloid β-protein (Aβ)-induced microglial activation^85^. A *CD40* knockout mouse model exhibits abnormal dendrite size and morphology^86^. Taken together, several loci that have been implicated to be underlying genetic loci for ADHD reached the stringent GWS threshold for the first time in our study by a multi-trait joint analysis approach which boosted study power by incorporating information for association with the other correlated traits.

Though we did not find an appropriate replication cohort for the loci identified in MTAG analysis of BIP and MDD, the biological relevance and the functional annotations of the candidate genes at these loci suggest that they are *bona fide* associations with these neuropsychiatric disorders. *TMEM56*, which was the nearest gene of SNP rs12563424 at the novel BIP locus 20q13.12 showed significant differential gene expression in some neurological disorders. TMEM56 was downregulated in the cerebellum with *Atxn2* knockout^87^ and was upregulated in nucleus accumbens in the maternal immune activation model of neurodevelopmental disorders^88^. SNP rs12563424 was also reported to be significantly associated with BIP at a conditional false-discovery rate (FDR) in a recent study^89^. *SPTBN2*, a candidate gene at the locus of MDD, is involved in the regulation of the glutamate signaling pathway and mutations in this gene result in a class of spinocerebellar ataxia with the feature of neurodegeneration^90^.

Among the candidate genes at the newly identified loci, several functions in immune regulation, suggesting the contribution of these pathways to the pathogenesis of neuropsychiatric disorders. The impact of dysregulated immune function on the development of neuropsychiatric disorders has been implicated in previous studies. For example, Tiosano *et al*. reported that there is a positive association between SCZ and SLE, a prototypic autoimmune disease, across patients of different ages, gender, and socioeconomic status, which suggests an overlap in genetic basis and molecular mechanism between SLE and SCZ^91^. Epidemiological evidence and animal models implicate maternal infections and activation of the maternal immune system in neuropathology in the fetus which can further impair lifelong neuropathology and result in altered behavior in offspring, making maternal infection during pregnancy a risk factor for neuropsychiatric disorders^92^.

Both common SNPs and rare CNVs have been implicated in the pathogenesis of complex human diseases, especially neuropsychiatric disorders. CNV studies revealed CNVs in neurodevelopmental genes confer increased risk to various neuropsychiatric disorders^93,94^. As we discussed above, many common variants have been associated with these diseases though usually with a much smaller odds ratio compared to rare CNVs. Overlaps in genes between common variants and rare CNVs have been seen, such as the 11p13.12 locus containing the *GRM5* gene. It would therefore be interesting to investigate the relationship between the rare CNVs and the common variants associated with the same disease to determine if the common variants or the haplotype defined by common variants are tagging for the rare CNVs, or if the SNPs confer synthetic effects mimicking the effects of the CNVs. It is also plausible that the common variants may serve as genetic modifiers for rare CNVs.

In our study, we increased study power by conducting joint analyses of correlated traits of neuropsychiatric disorders, and further performed a replication study of ADHD using a pediatric cohort, which provides the advantage of studying an early onset neuropsychiatric disease. However, the pediatric cohort also has two major limitations. It cannot be an ideal replication for certain other neuropsychiatric disorders with the age of onset above 20 years of age and with a different spectrum of clinical features, and the sample size of our replication cohort is small compared to the discovery PGC cohort. In our ADHD replication study, none of the five loci passed the multiple-testing adjusted threshold of *P* value < 0.01. For common variants with small effect sizes, the size of the replication cohort limits the ability to fully recapitulate the associations found in the discovery cohort. Failure to achieve corrected significance also reflects the heterogeneity between studies, which could arise from the patients’ characteristics or the different phenotyping and cohort ascertainment between cohorts. However, sign test and correlation test of all GWS associations, as well as the genetic correlation based on genome-wide SNP data, suggest the overall consistency between the ADHD MTAG analysis and our pediatric cohort which provides replication of nominal significance. The other novel loci found in our study are also highly biologically relevant and we anticipate that more of them will be replicated in future studies.

In conclusion, through meta-analysis and multi-trait joint analyses of GWAS data of five neuropsychiatric disorders and an independent replication analysis, multiple novel genetic loci were identified with a predisposition to neuropsychiatric disorders. Furthermore, the causal variants at these loci may regulate target gene expression at least in part by affecting histone modifications at various brain regions. Taken together, our research provides novel insight into the genetic basis of neuropsychiatric disorders which may uncover new therapeutic opportunities and provide a molecular basis for drug repositioning.

## Supplementary information

Supplementary material

Supplementary Table 2

Supplementary Table 3

Supplementary Table 4

Supplementary Table 5

Supplementary Table 6

Supplementary Table 7

Supplementary Table 8

Supplementary Table 9

Supplementary Table 10

Supplementary Table 11

Supplementary Table 12

Supplementary Table 13

Supplementary Table 14

## Data Availability

To have access to the CAG replication cohort, please contact the corresponding author Dr. Hakon Hakonarson.

## Code availability

All analyses followed the standard pipeline of MTAG, METAL, and GWAS analyses and all the scripts are available from the authors upon request.
