# Supplementary material for: Integrative analysis of genome-wide association studies identifies novel loci associated with neuropsychiatric disorders
Source: Transl Psychiatry. 2021 Jan 21;11:69. doi: 10.1038/s41398-020-01195-5 (PMC7820351; doi:10.1038/s41398-020-01195-5)

Supplementary material

Supplementary Table 1. The five cohorts of European ancestry for the five neuropsychiatric disorders downloaded from the website of Psychiatric Genomics Consortium (PGC).

Disease	Case	Control	Cohort name	PMID
ADHD	19099	34194	European-Ancestry-GWAS	30478444
ASD	18381	27969	ASD iPSYCH – PGC GWAS – 2017 (publ. 2019)	30804558
BIP	20352	31358	BIP 2018	31043756
MDD	170756	329443	2019 PGC UKB Depression Genome-wide	30718901
SCZ	33640	43456	EUR	31740837

Disease= each neuropsychiatric disorder in the study, ADHD= attention deficit hyperactivity disorder, ASD= autism spectrum disorder, BIP= bipolar disorder, MDD= major depressive disorder, SCZ= schizophrenia; Case= the number of cases in each study; Control= the number of controls in each study; Cohort name= the name of the link to the GWAS cohort on PGC website; PMID= PubMed Unique Identifier of the original publication of each cohort.

Supplementary Figure 1. Quantile-quantile (QQ) plot of summary statistics for multi-trait joint analysis and meta-analysis of five neuropsychiatric diseases: MTAG analysis of ADHD (**A**), ASD (**B**), BIP (**C**), MDD (**D**), SCZ (**E**), and METAL analysis of five disorders (**F**). The standardized genomic inflation factor of each analysis was indicated in the figure.

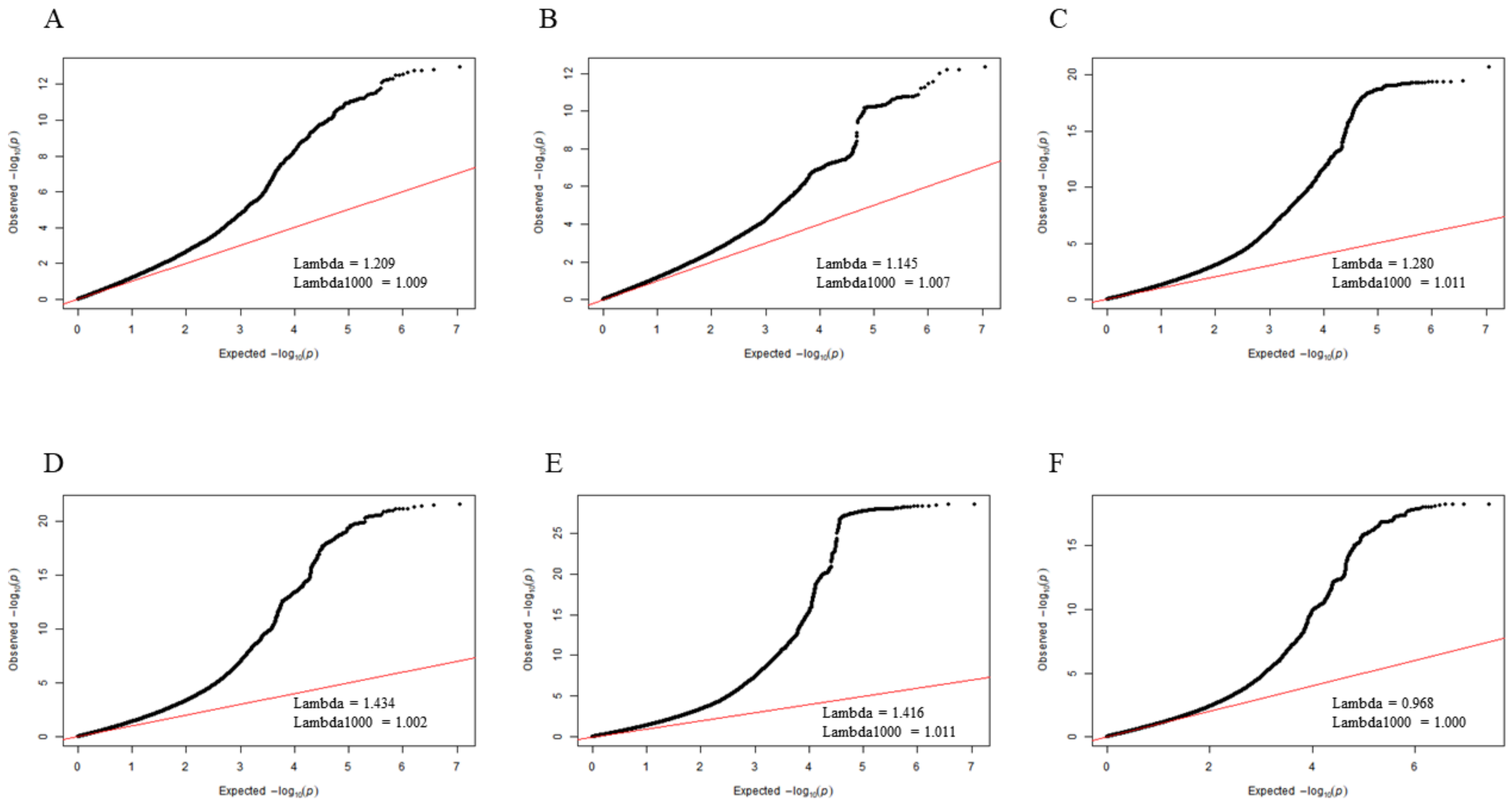

Supplementary Figure 2. Regional association plots of novel significant loci from multi-trait joint analyses.

Regional association plots showing the novel significant loci identified from multi-trait joint analyses of ADHD (**A**), BIP (**B**) and MDD (**C**). The genomic position of each SNP is shown on the X-axis and the $-\log_{10}$ (P-values) are shown on the Y-axis. The most significant SNP is indicated in purple color and the rest of the SNPs are shown in different colors corresponding to the linkage disequilibrium with the top associated SNP. The blue vertical line indicates the recombination rate based on the HapMap project.

A

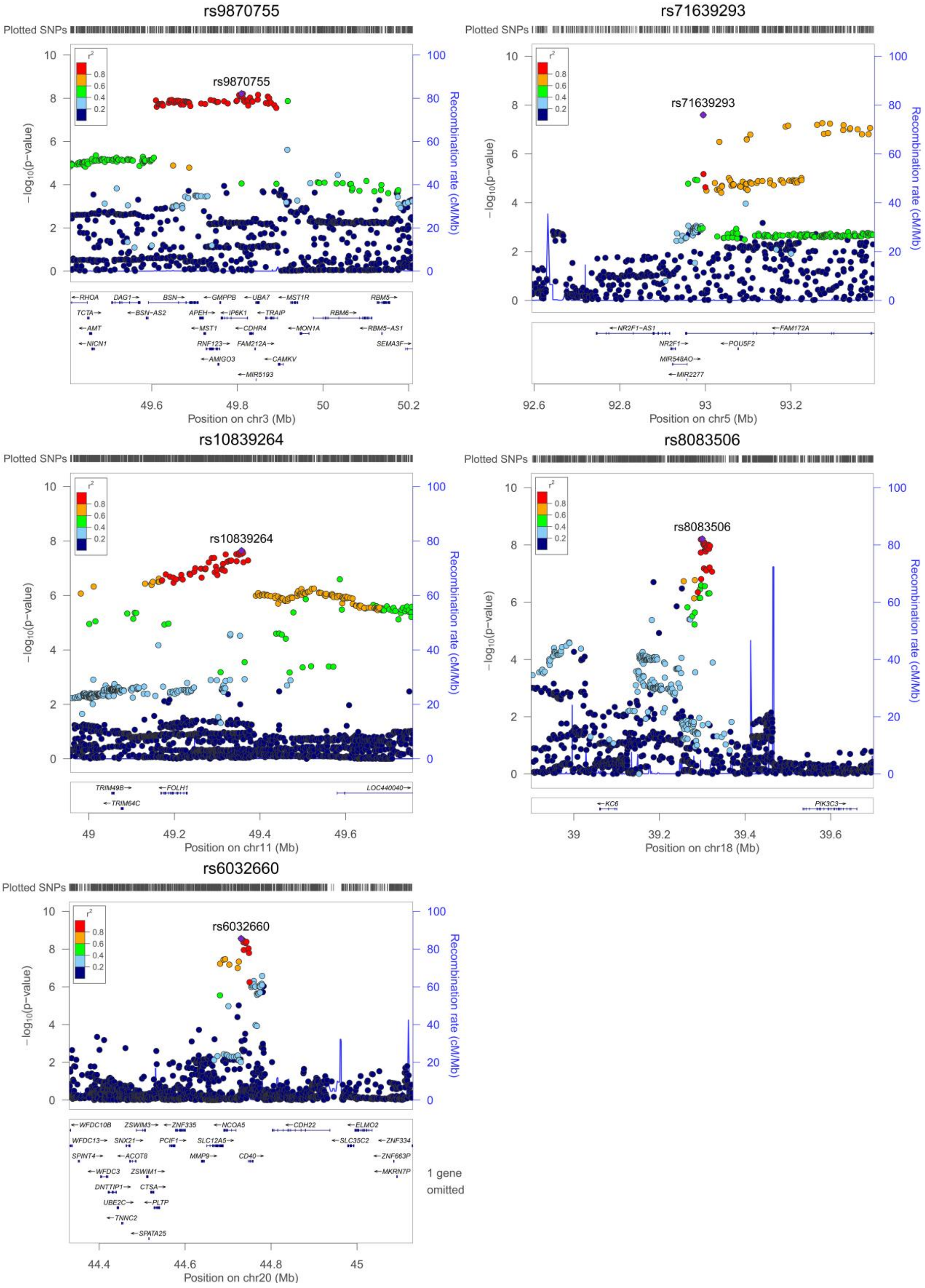

rs9870755
Plotted SNPs
−log10(p−value)
Recombination rate (cM/Mb)
Position on chr3 (Mb)
RHOA
DAG1
BSN
GMPPB
UBA7
MST1R
RBM5
TCTA
BSN−AS2
APEH
IP6K1
TRAIP
RBM6
AMT
MST1
CDHR4
MON1A
RBM5−AS1
NICN1
RNF123
FAM212A
SEMA3F
AMIGO3
CAMKV
MIR5193
rs71639293
Position on chr5 (Mb)
NR2F1−AS1
FAM172A
NR2F1
POU5F2
MIR548AO
MIR2277
rs10839264
Position on chr11 (Mb)
TRIM49B
FOLH1
LOC440040
TRIM64C
rs8083506
Position on chr18 (Mb)
KC6
PIK3C3
rs6032660
Position on chr20 (Mb)
WFDC10B
ZSWIM3
ZNF335
NCOA5
CDH22
ELMO2
WFDC13
SNX21
PCIF1
SLC12A5
SLC35C2
ZNF334
SPINT4
ACOT8
MMP9
CD40
ZNF663P
WFDC3
ZSWIM1
MKRN7P
DNTTIP1
CTSA
UBE2C
PLTP
TNNC2
SPATA25
1 gene omitted

B

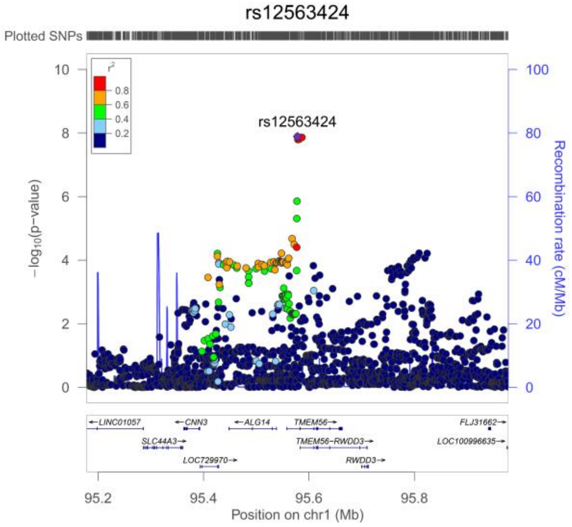
rs12563424
Plotted SNPs
rs12563424
−log10(p−value)
Recombination rate (cM/Mb)
r²
0.8
0.6
0.4
0.2
LINC01057
CNN3
ALG14
TMEM56
FLJ31662
SLC44A3
TMEM56−RWDD3
LOC100996635
LOC729970
RWDD3
95.2
95.4
95.6
95.8
Position on chr1 (Mb)

C

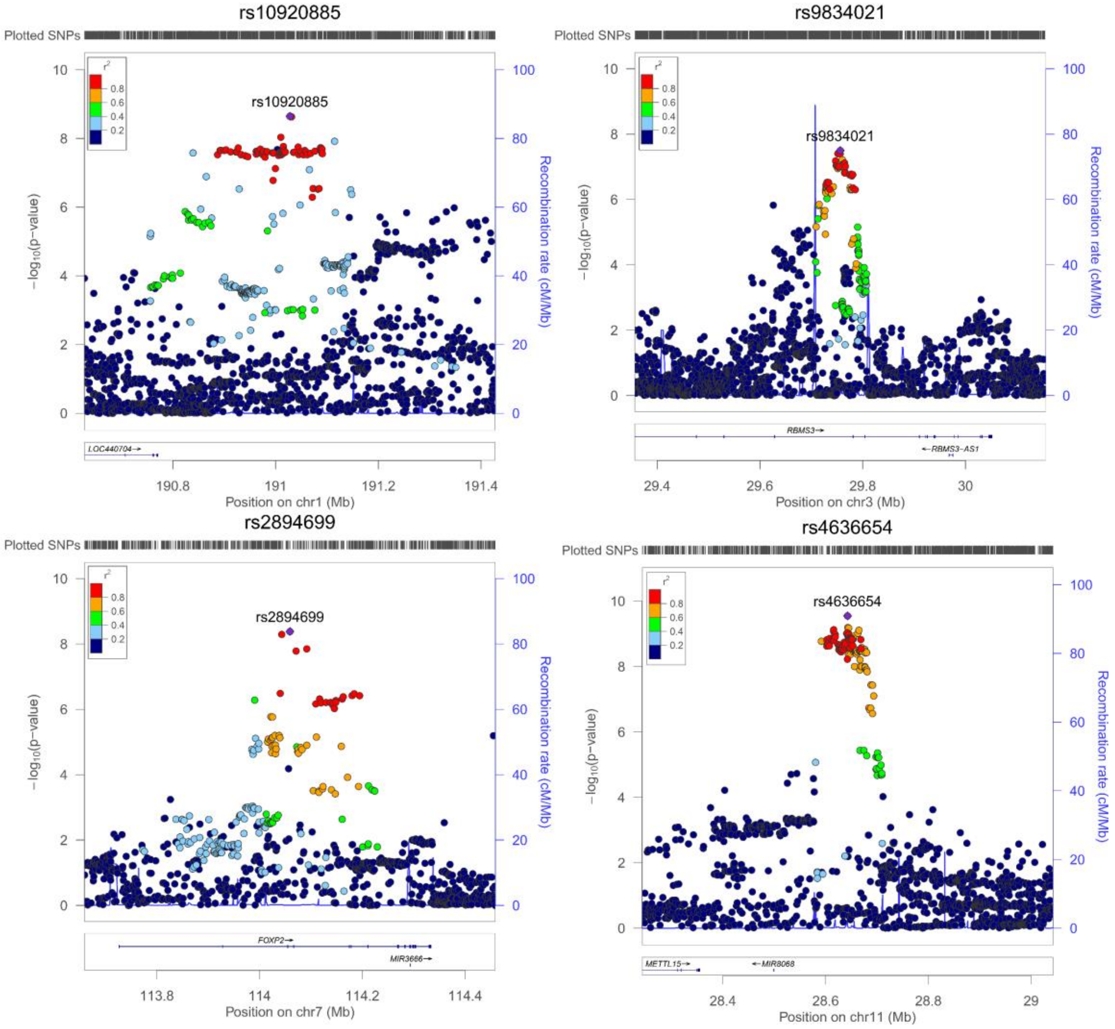
rs10920885
Plotted SNPs
rs10920885
−log10(p−value)
Recombination rate (cM/Mb)
LOC440704
190.8
191
191.2
191.4
Position on chr1 (Mb)
rs9834021
Plotted SNPs
rs9834021
RBMS3
RBMS3−AS1
29.4
29.6
29.8
30
Position on chr3 (Mb)
rs2894699
Plotted SNPs
rs2894699
FOXP2
MIR3666
113.8
114
114.2
114.4
Position on chr7 (Mb)
rs4636654
Plotted SNPs
rs4636654
METTL15
MIR8068
28.4
28.6
28.8
29
Position on chr11 (Mb)

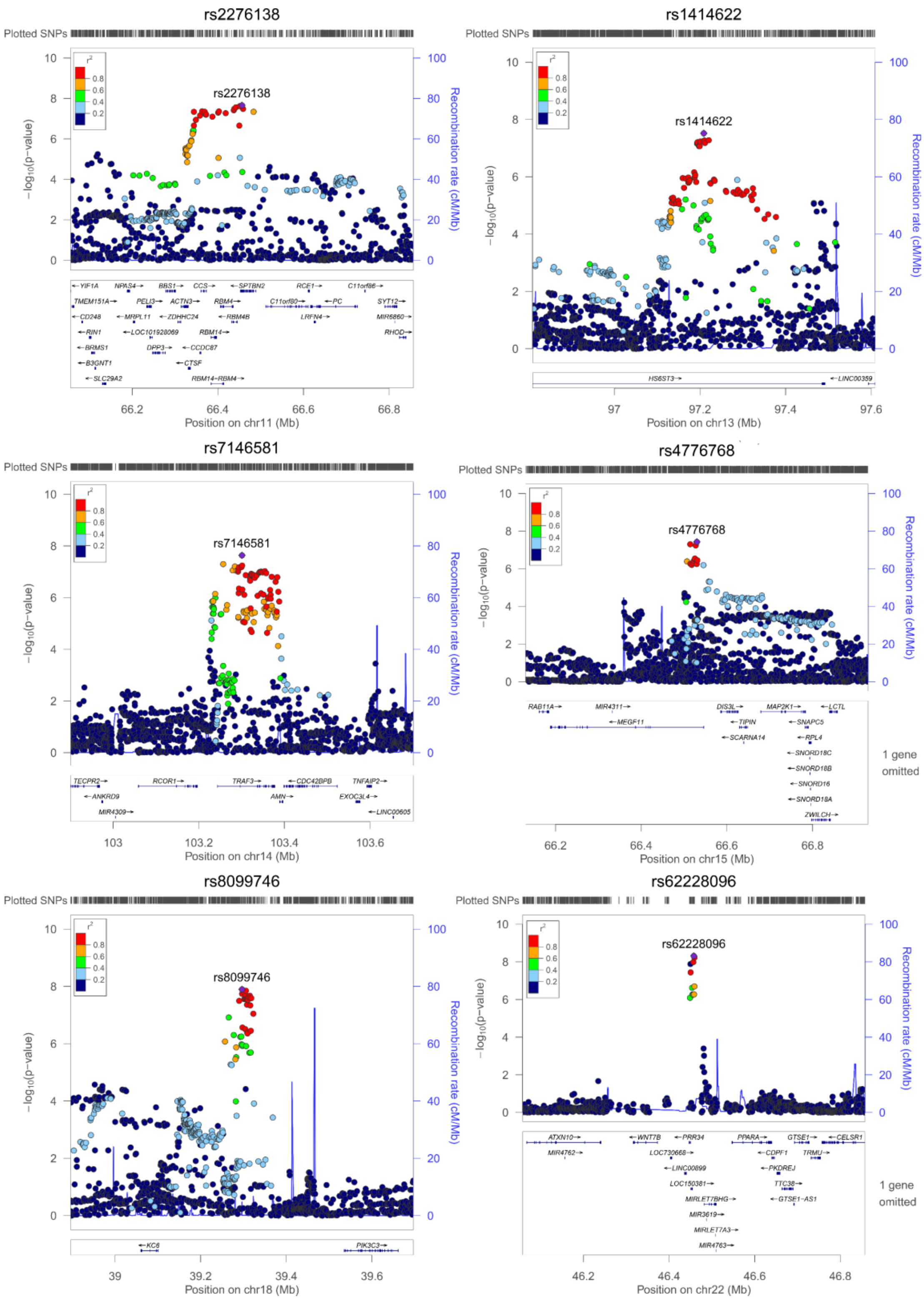

rs2276138
Plotted SNPs
−log10(p−value)
Recombination rate (cM/Mb)
Position on chr11 (Mb)
YIF1A
NPAS4
BBS1
CCS
SPTBN2
RCE1
C11orf86
TMEM151A
PELI3
ACTN3
RBM4
C11orf80
PC
SYT12
CD248
MRPL11
ZDHHC24
RBM4B
LRFN4
MIR6860
RIN1
LOC101928069
RBM14
RHOD
BRMS1
DPP3
CCDC87
B3GNT1
CTSF
SLC29A2
RBM14−RBM4
rs1414622
Position on chr13 (Mb)
HS6ST3
LINC00359
rs7146581
Position on chr14 (Mb)
TECPR2
RCOR1
TRAF3
CDC42BPB
TNFAIP2
ANKRD9
AMN
EXOC3L4
MIR4309
LINC00605
rs4776768
Position on chr15 (Mb)
RAB11A
MIR4311
DIS3L
MAP2K1
LCTL
MEGF11
TIPIN
SNAPC5
SCARNA14
RPL4
SNORD18C
SNORD18B
SNORD16
SNORD18A
ZWILCH
1 gene omitted
rs8099746
Position on chr18 (Mb)
KC6
PIK3C3
rs62228096
Position on chr22 (Mb)
ATXN10
WNT7B
PRR34
PPARA
GTSE1
CELSR1
MIR4762
LOC730668
CDPF1
TRMU
LINC00899
PKDREJ
LOC150381
TTC38
MIRLET7BHG
GTSE1−AS1
MIR3619
MIRLET7A3
MIR4763
1 gene omitted

Supplementary Figure 3. The forest plots of index SNP at each novel locus demonstrating the P value, log odds ratio (beta) and standard error for each SNP in each disease specific association testing. (**A**) ADHD; (**B**) BIP; (**C**) MDD.

A

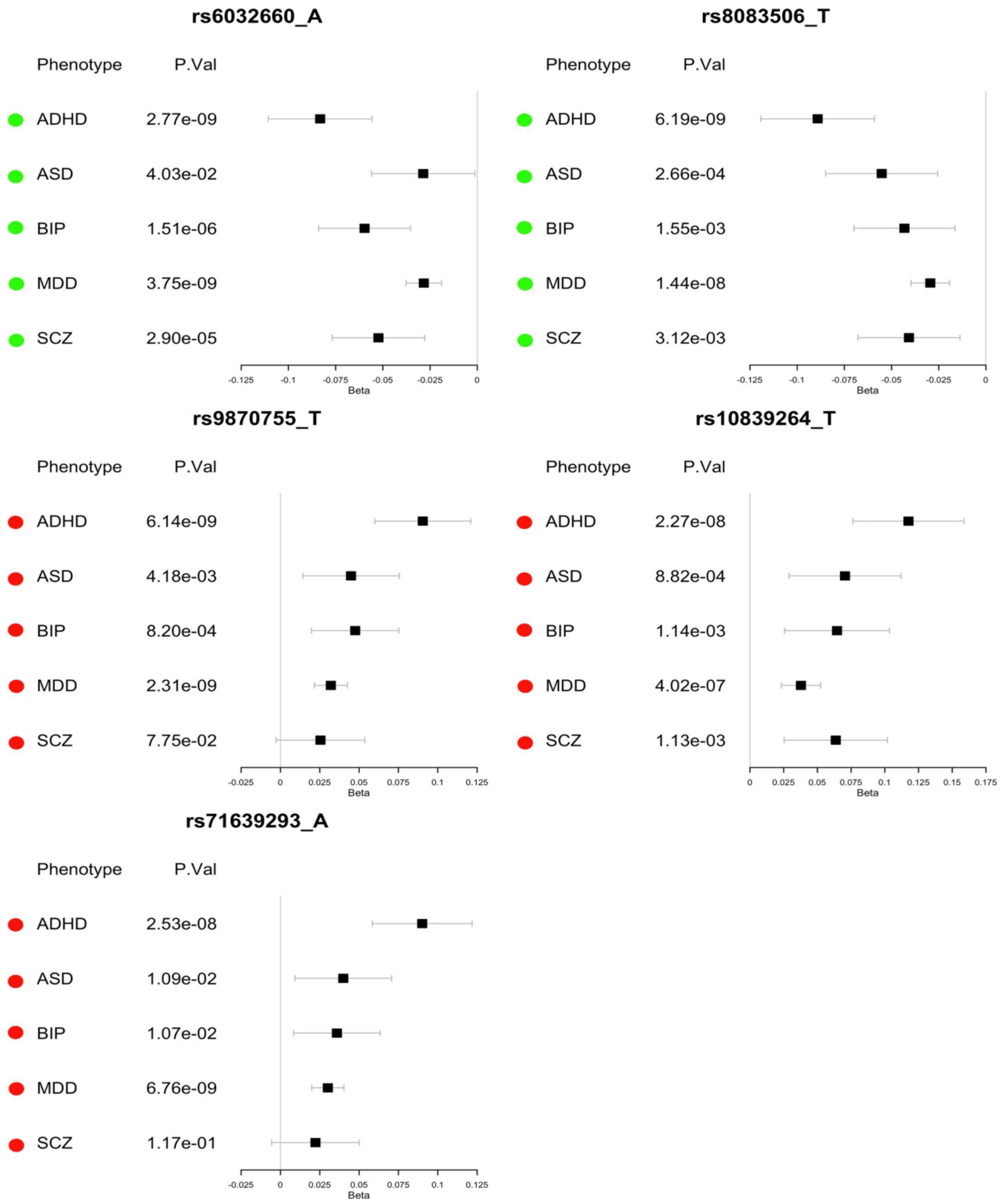

B

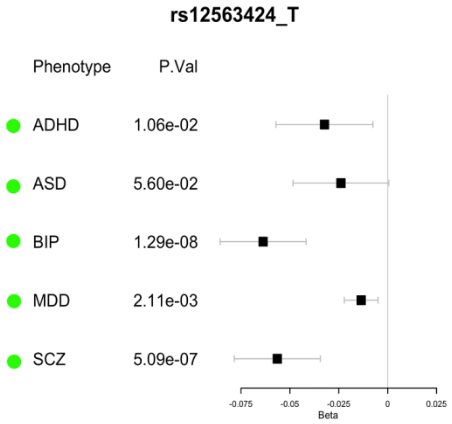
rs12563424_T
Phenotype
P.Val
ADHD
1.06e-02
ASD
5.60e-02
BIP
1.29e-08
MDD
2.11e-03
SCZ
5.09e-07
-0.075
-0.05
-0.025
0
0.025
Beta

C

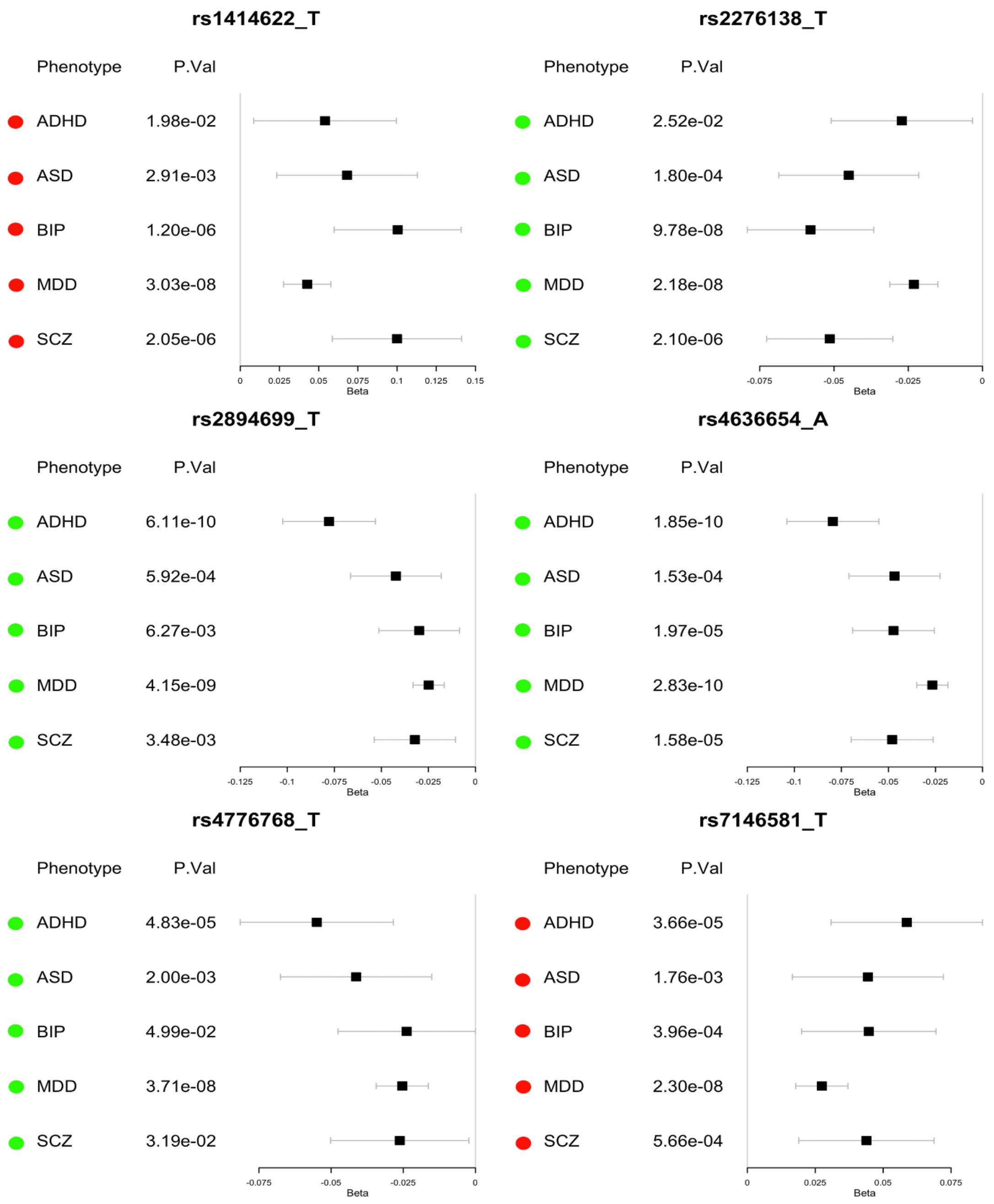
rs1414622_T
Phenotype P.Val
ADHD 1.98e-02
ASD 2.91e-03
BIP 1.20e-06
MDD 3.03e-08
SCZ 2.05e-06
0 0.025 0.05 0.075 0.1 0.125 0.15
Beta
rs2276138_T
Phenotype P.Val
ADHD 2.52e-02
ASD 1.80e-04
BIP 9.78e-08
MDD 2.18e-08
SCZ 2.10e-06
-0.075 -0.05 -0.025 0
Beta
rs2894699_T
Phenotype P.Val
ADHD 6.11e-10
ASD 5.92e-04
BIP 6.27e-03
MDD 4.15e-09
SCZ 3.48e-03
-0.125 -0.1 -0.075 -0.05 -0.025 0
Beta
rs4636654_A
Phenotype P.Val
ADHD 1.85e-10
ASD 1.53e-04
BIP 1.97e-05
MDD 2.83e-10
SCZ 1.58e-05
-0.125 -0.1 -0.075 -0.05 -0.025 0
Beta
rs4776768_T
Phenotype P.Val
ADHD 4.83e-05
ASD 2.00e-03
BIP 4.99e-02
MDD 3.71e-08
SCZ 3.19e-02
-0.075 -0.05 -0.025 0
Beta
rs7146581_T
Phenotype P.Val
ADHD 3.66e-05
ASD 1.76e-03
BIP 3.96e-04
MDD 2.30e-08
SCZ 5.66e-04
0 0.025 0.05 0.075
Beta

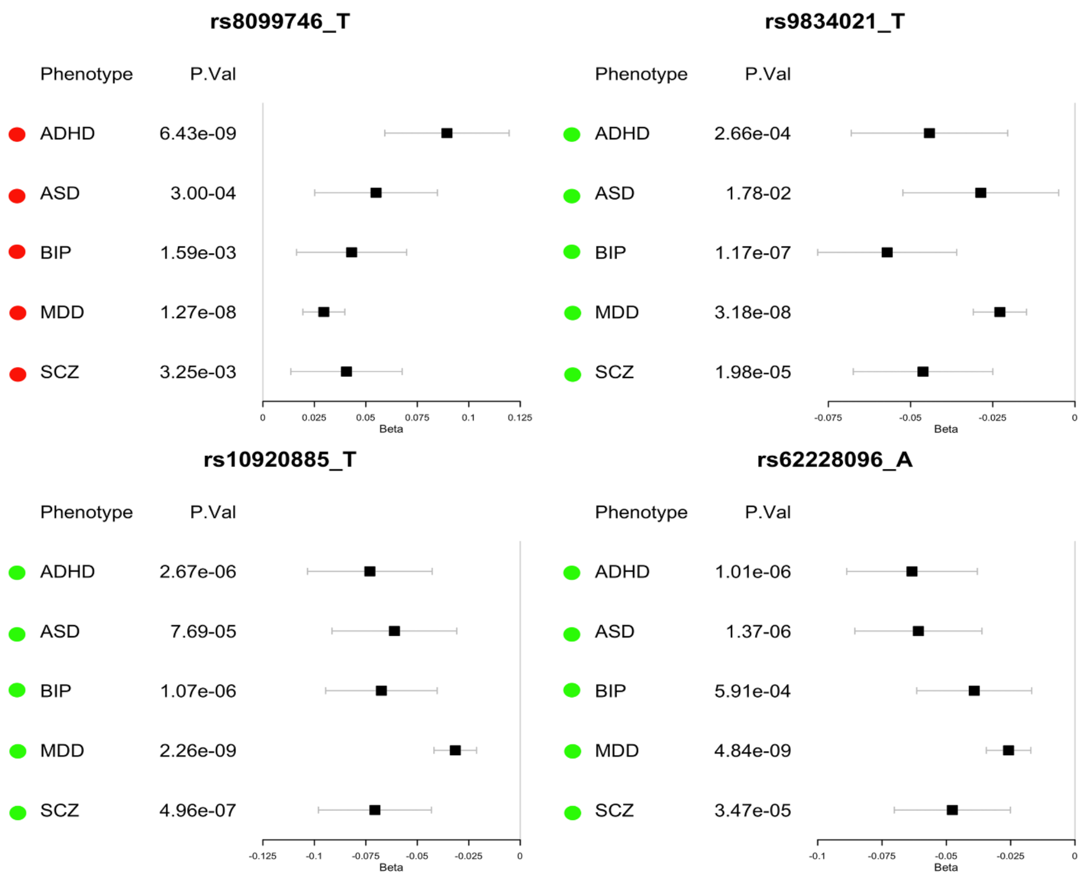
rs8099746_T
Phenotype
P.Val
ADHD
6.43e-09
ASD
3.00-04
BIP
1.59e-03
MDD
1.27e-08
SCZ
3.25e-03
0
0.025
0.05
0.075
0.1
0.125
Beta
rs9834021_T
Phenotype
P.Val
ADHD
2.66e-04
ASD
1.78-02
BIP
1.17e-07
MDD
3.18e-08
SCZ
1.98e-05
-0.075
-0.05
-0.025
0
Beta
rs10920885_T
Phenotype
P.Val
ADHD
2.67e-06
ASD
7.69-05
BIP
1.07e-06
MDD
2.26e-09
SCZ
4.96e-07
-0.125
-0.1
-0.075
-0.05
-0.025
0
Beta
rs62228096_A
Phenotype
P.Val
ADHD
1.01e-06
ASD
1.37-06
BIP
5.91e-04
MDD
4.84e-09
SCZ
3.47e-05
-0.1
-0.075
-0.05
-0.025
0
Beta

Supplementary Figure 4. Regional association plot of the novel significant locus on chromosome 22 in the meta-analysis of five neuropsychiatric disorders.

The regional association plot was generated using the LocusZoom software. The genomic coordinates are plotted on the axis, and the –log10 P-values of SNPs are shown on the Y-axis. The most significant SNP is indicated in purple and the remaining SNPs are shown in different colors based on linkage disequilibrium with top associated SNP (the color coding legend is indicated in the upper corner of each plot). The blue vertical line indicates the recombination rate based on the HapMap project.

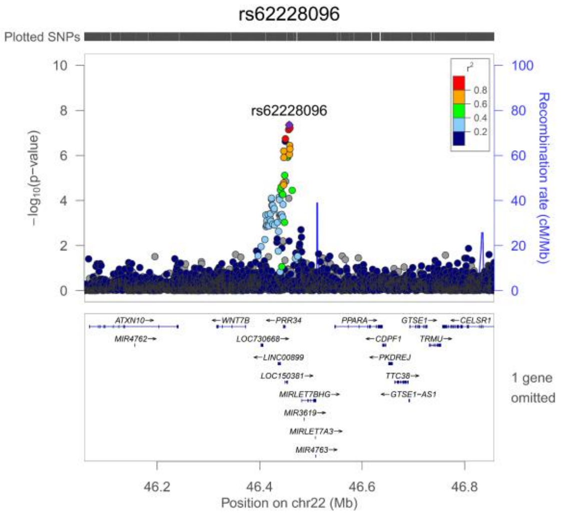

Supplementary Figure 5. Circos plots showing chromatin interactions between causal variants and distant genes.

Each causal variant is indicated on the most outer layer. Y-axis are ranged between 0 to the maximum -log10(P-value) of the SNPs. The further the SNP is from the center of the circle, the smaller the MTAG P-value is. The dark blue regions on the chromosome ring of the second layer and that of the third layer display the genomic locus where each causal variant is located at. The mapped genes by either chromatin interaction and/or eQTLs are shown on the chromosome ring of the third layer and the links display the chromatin interactions. Genes mapped only by chromatin interactions or only by eQTLs are colored orange or green, respectively. When the gene is mapped by both, it is colored red. (**A**) ADHD; (**B**) BIP; (**C**) MDD.

A

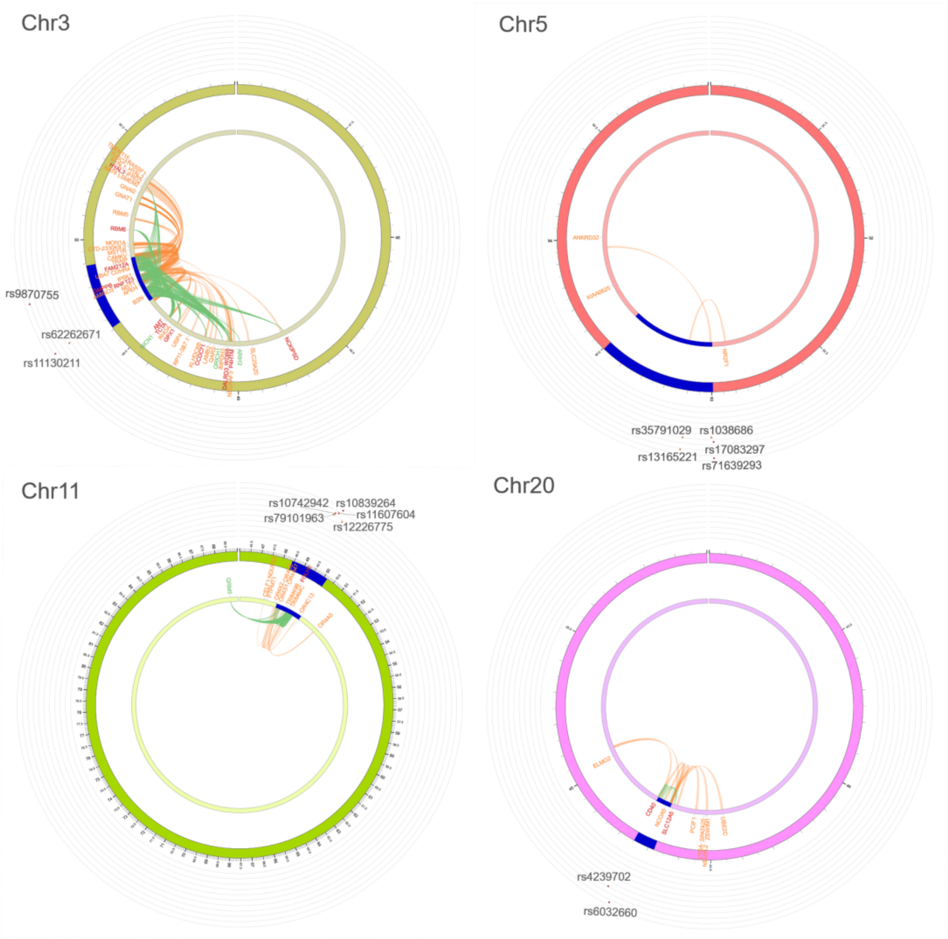

B

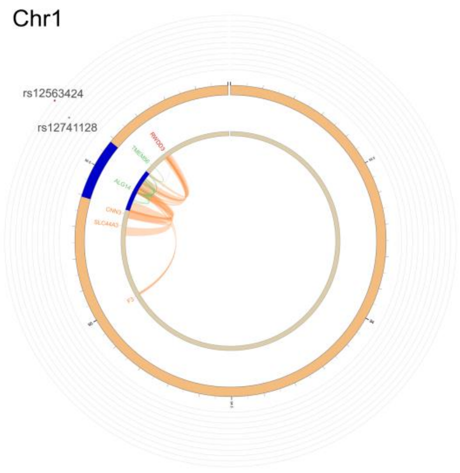
Chr1
rs12563424
rs12741128

C

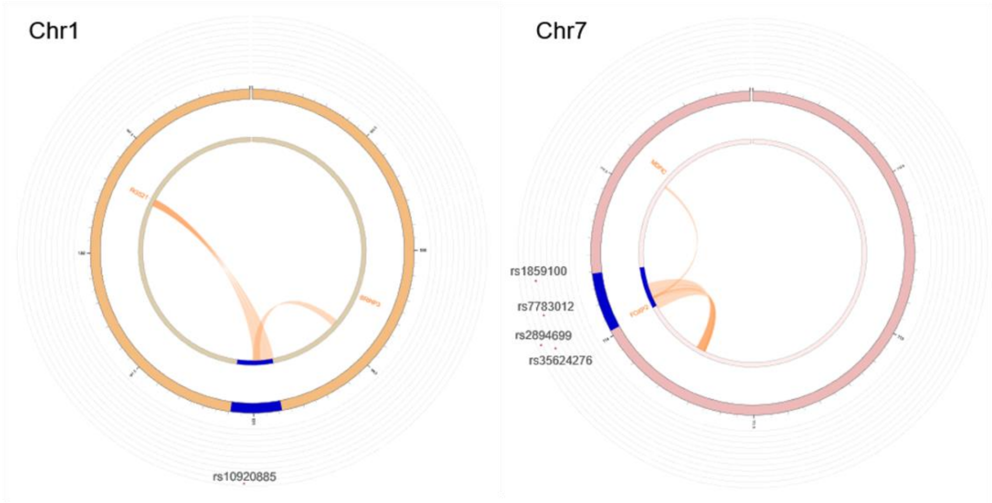
Chr1
rs10920885
Chr7
rs1859100
rs7783012
rs2894699
rs35624276

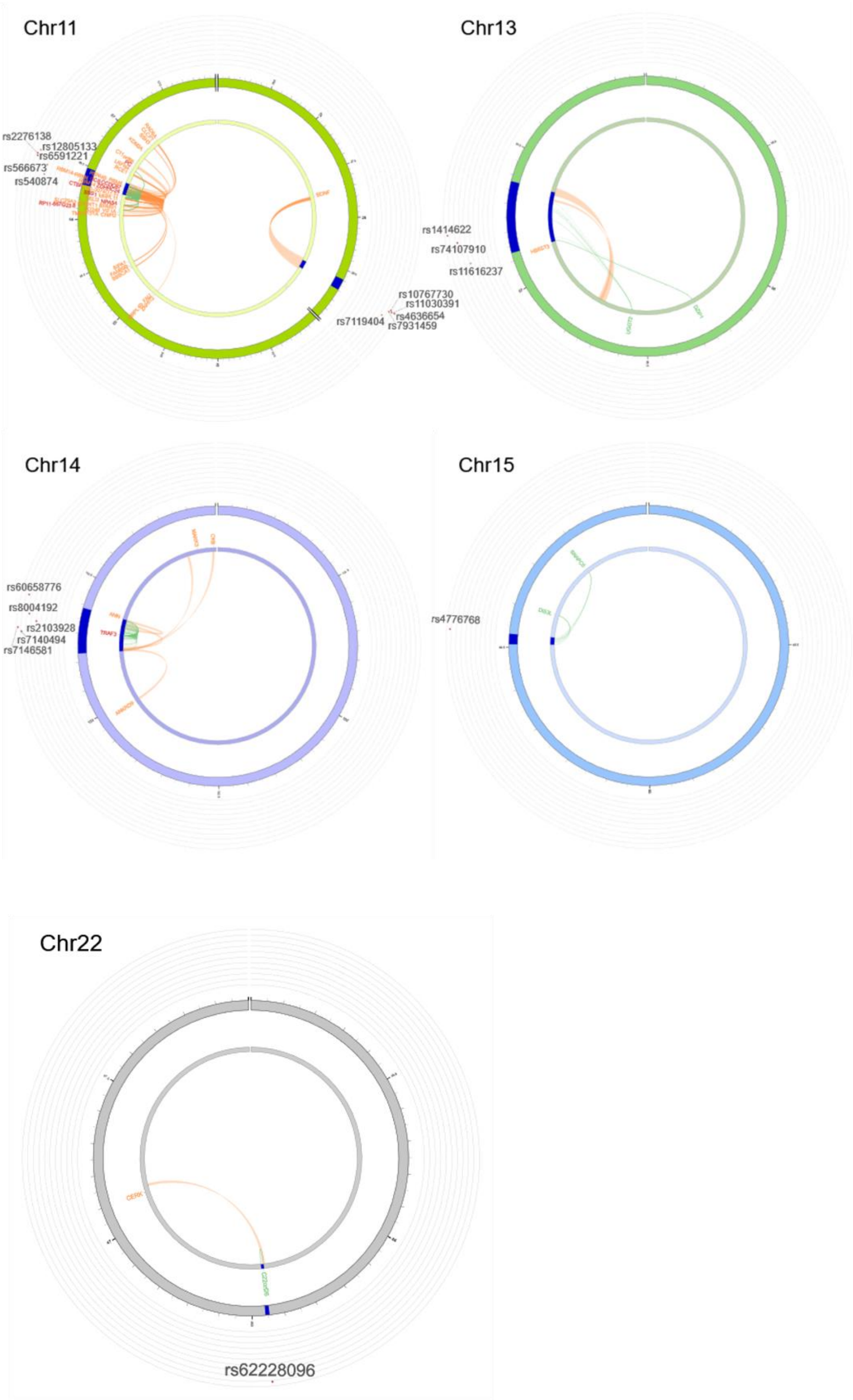
Chr11
rs2276138
rs12805133
rs6591221
rs566673
rs540874
rs10767730
rs11030391
rs4636654
rs7931459
rs7119404
Chr13
rs1414622
rs74107910
rs11616237
Chr14
rs60658776
rs8004192
rs2103928
rs7140494
rs7146581
Chr15
rs4776768
Chr22
rs62228096

Supplementary Figure 6. MAGMA gene expression profile analysis of genes at novel loci identified in MTAG analyses of neuropsychiatric disorders. MAGMA is a generalized gene-set analysis approach, here showing the differentially expressed gene sets **A**) at 29 different ages of human brain samples from BrainSpan.; **B**) at 11 general developmental stages of brain samples from BrainSpan. The ages or developmental stages with significant enrichment of differentially expressed gene sets were colored red. yrs = years; pcw = post conception weeks.

A

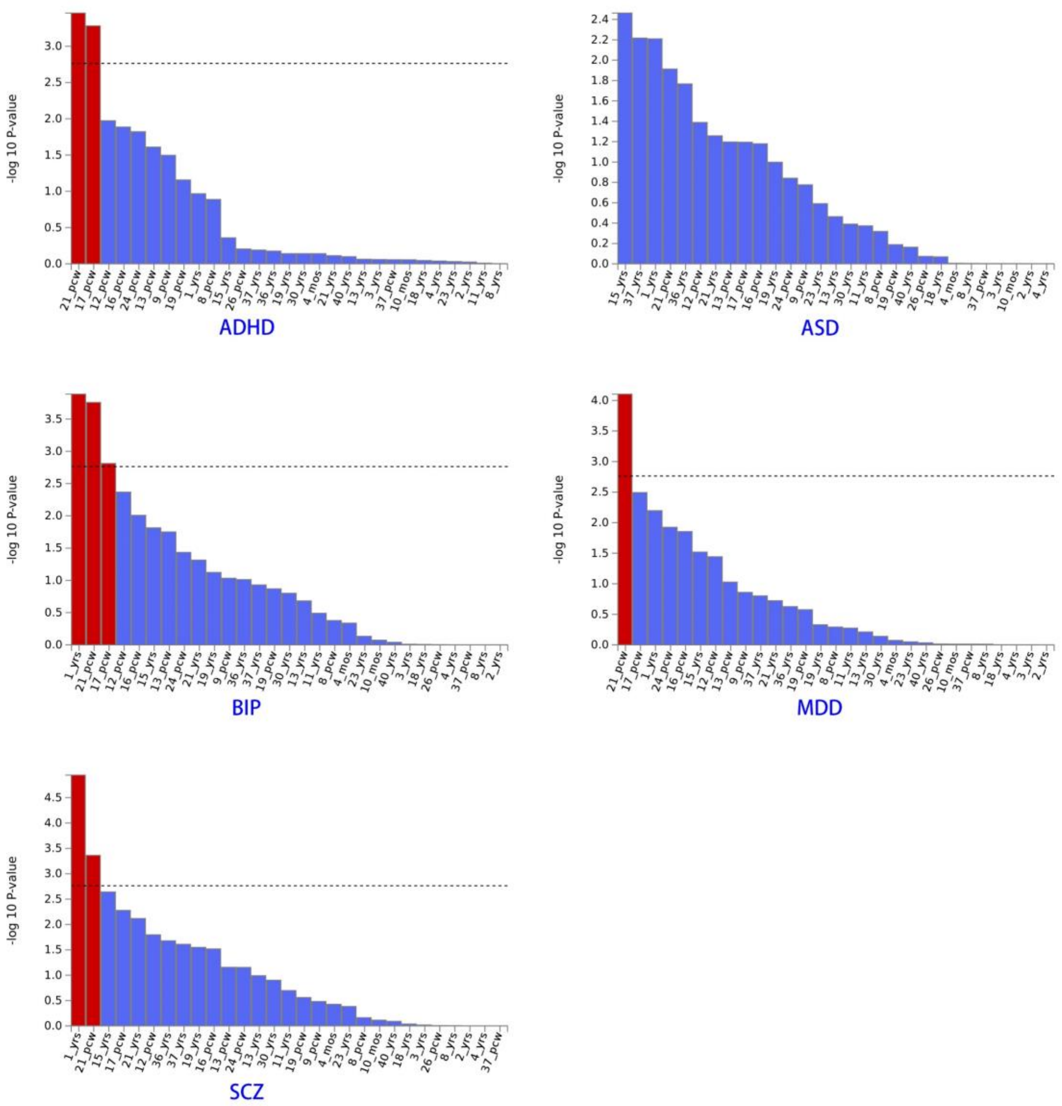

B

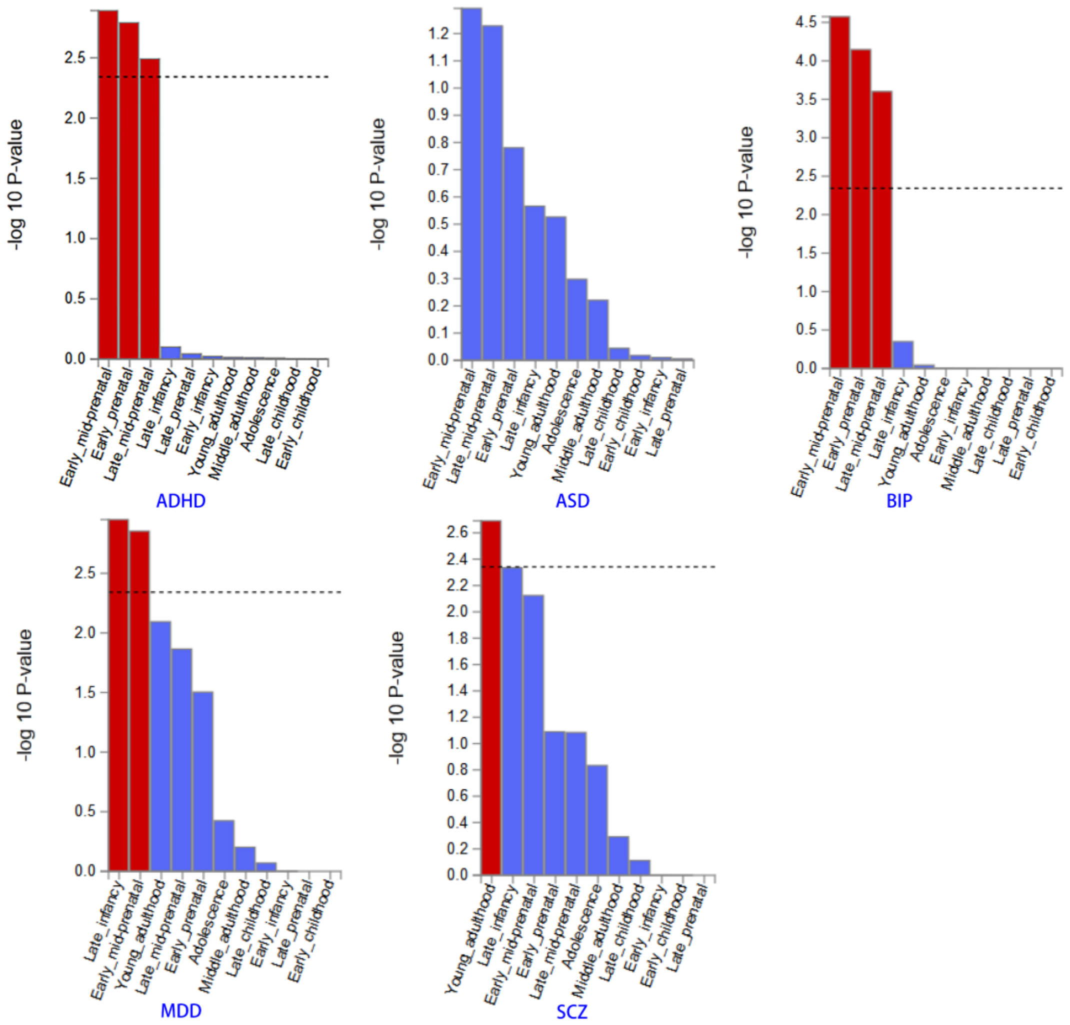
-log 10 P-value
0.0
0.5
1.0
1.5
2.0
2.5
Early_mid-prenatal
Early_prenatal
Late_mid-prenatal
Late_infancy
Late_prenatal
Early_infancy
Young_adulthood
Middle_adulthood
Adolescence
Late_childhood
Early_childhood
ADHD
-log 10 P-value
0.0
0.1
0.2
0.3
0.4
0.5
0.6
0.7
0.8
0.9
1.0
1.1
1.2
Early_mid-prenatal
Late_mid-prenatal
Early_prenatal
Late_infancy
Young_adulthood
Adolescence
Middle_adulthood
Late_childhood
Early_childhood
Early_infancy
Late_prenatal
ASD
-log 10 P-value
0.0
0.5
1.0
1.5
2.0
2.5
3.0
3.5
4.0
4.5
Early_mid-prenatal
Early_prenatal
Late_mid-prenatal
Late_infancy
Young_adulthood
Adolescence
Early_infancy
Middle_adulthood
Late_childhood
Late_prenatal
Early_childhood
BIP
-log 10 P-value
0.0
0.5
1.0
1.5
2.0
2.5
Late_infancy
Early_mid-prenatal
Young_adulthood
Late_mid-prenatal
Early_prenatal
Adolescence
Middle_adulthood
Late_childhood
Early_infancy
Late_prenatal
Early_childhood
MDD
-log 10 P-value
0.0
0.2
0.4
0.6
0.8
1.0
1.2
1.4
1.6
1.8
2.0
2.2
2.4
2.6
Young_adulthood
Late_infancy
Early_mid-prenatal
Early_prenatal
Late_mid-prenatal
Adolescence
Middle_adulthood
Late_childhood
Early_infancy
Early_childhood
Late_prenatal
SCZ

Supplementary Figure 7. Estimated heritability of five neuropsychiatric diseases. The liability scale SNP heritability based on MTAG results (MTAG) were compared with estimates from the original GWAS of each cohort (REF) and those from population studies reported in literature (POP). The population prevalence of each disease used in the heritability estimations is ADHD=0.05, ASD= 0.012, BIP=0.02, MDD= 0.302, SCZ= 0.01, which are the same as that in their original GWAS. The population heritability referred to the median value of heritability reported in prior studies in literature (**Supplementary Table 12**).

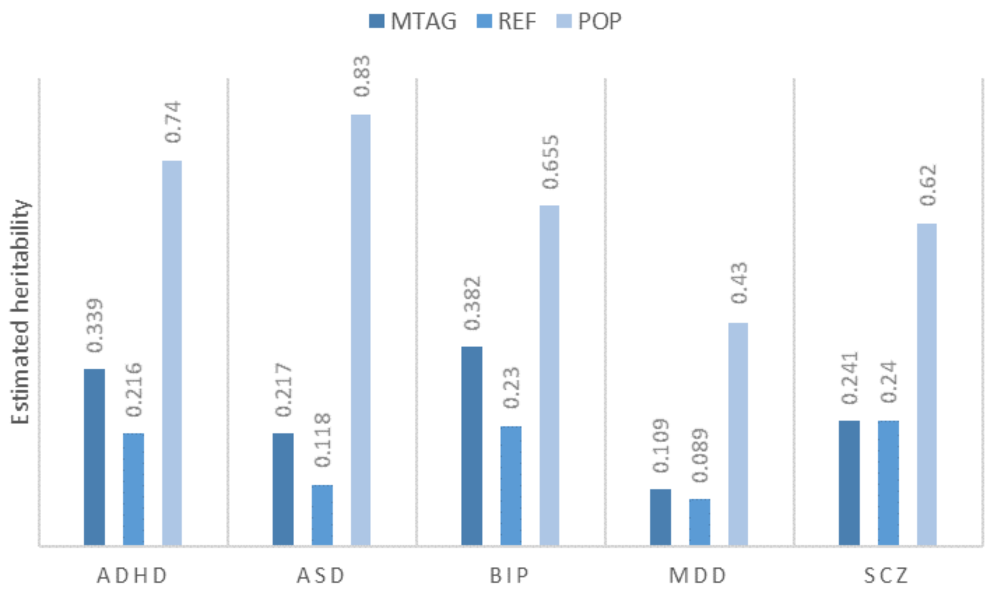

Supplementary Figure 8. Genetic correlation between the five neuropsychiatric disorders. The circles representing the five neuropsychiatric disorders, and the width of the line between the circles represents the strength of the pairwise genetic relationships in a positive correlation. All genetic correlations displayed in the schematic diagram were significant (P <0.05).

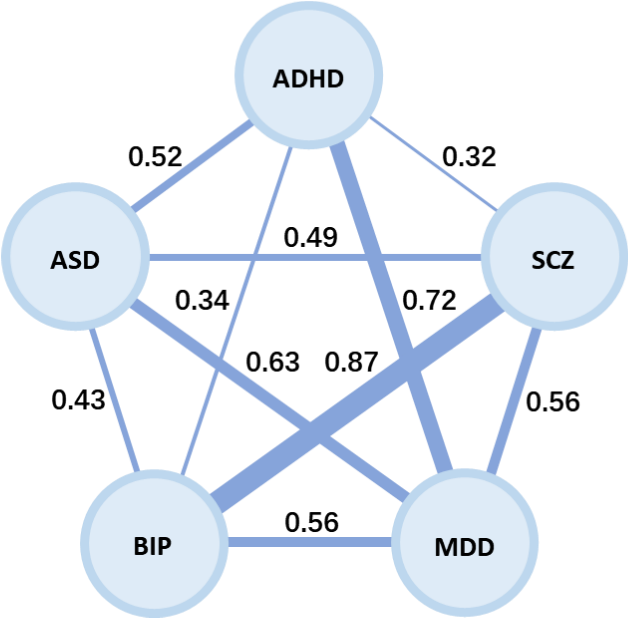

Supplementary Figure 9. The QQ-plot of the pediatric replication cohort.

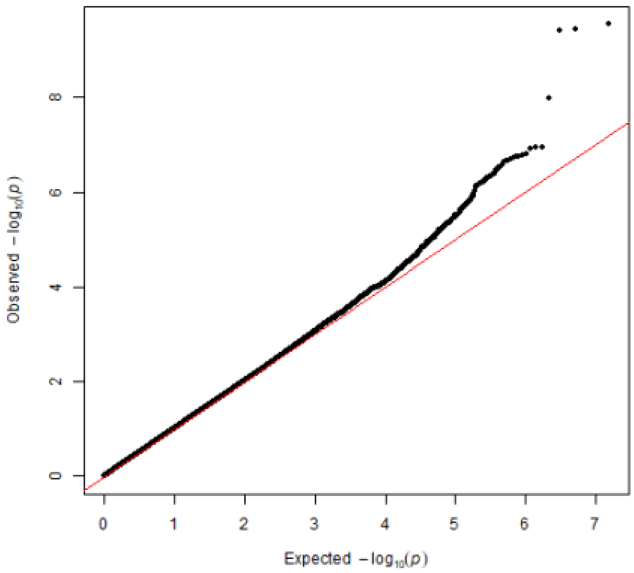

λ=1.013

Supplementary Figure 10. Regression plot showing test for correlation of effect size estimates at genome-wide significant loci from ADHD MTAG analysis of 19099 cases and 34194 controls after adjustment for winner's curse and those from the pediatric replication cohort of 2726 ADHD cases and 16321 controls. The effect size estimates from the ADHD MTAG analysis are shown on the X-axis and those from the pediatric replication cohort are indicated on the Y-axis. The blue vertical lines indicate the standard error of the effect size estimates. The color of each dot represents its -log10(p) from the ADHD MTAG analysis. The dashed purple line indicates the observed slope of the linear regression between the effect size estimates from the two studies.

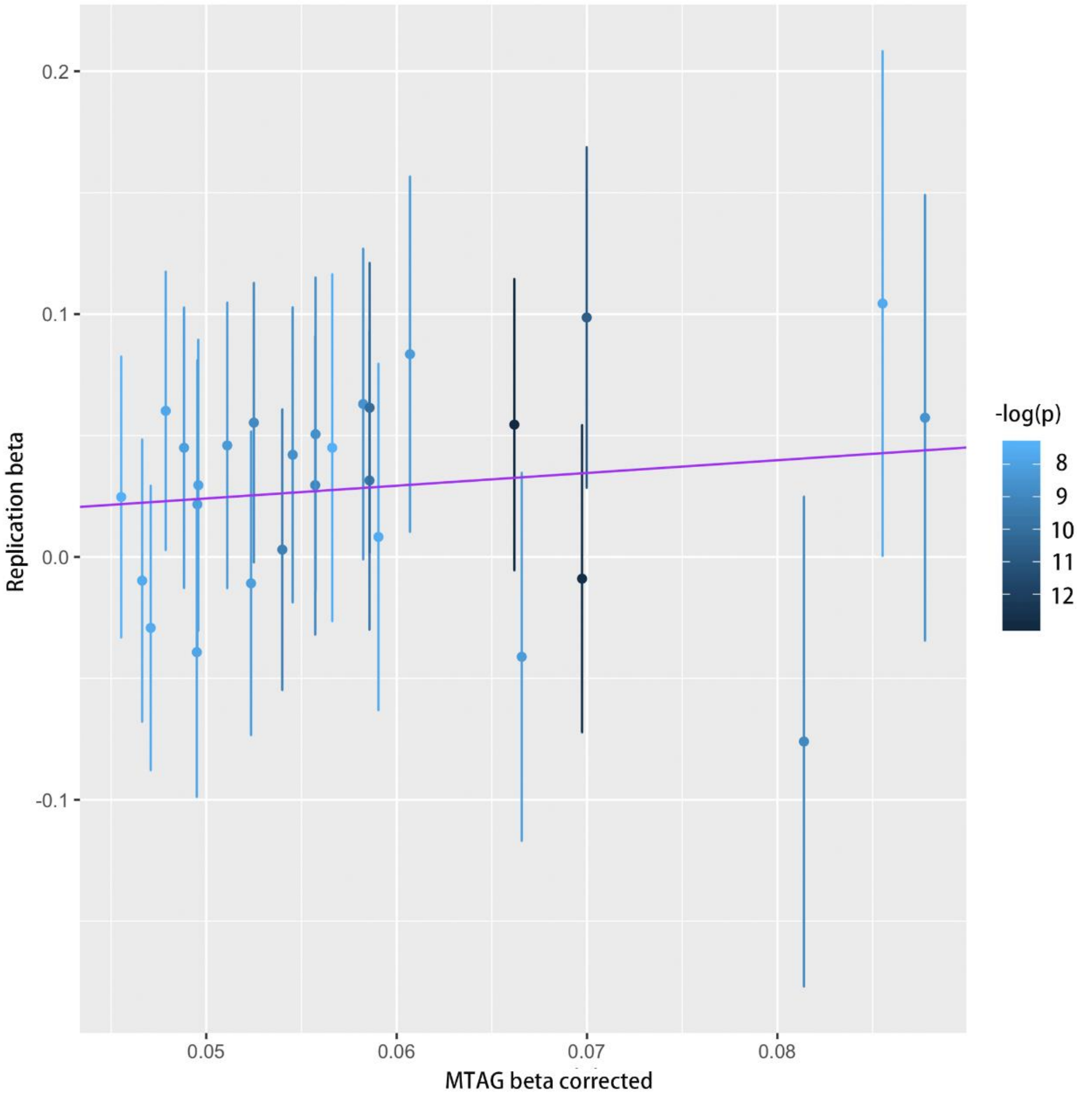

Supplement: Supplementary file 1 — Supplementary material [file 41398_2020_1195_MOESM1_ESM.pdf]
